# Flows of Substances in Networks and Network Channels: Selected Results and Applications

**DOI:** 10.3390/e24101485

**Published:** 2022-10-18

**Authors:** Zlatinka I. Dimitrova

**Affiliations:** Institute of Mechanics, Bulgarian Academy of Sciences, Acad. G. Bonchev Str., Bl. 4, 1113 Sofia, Bulgaria; zdim@imbm.bas.bg

**Keywords:** network, flow in a network, network channel, probability distribution, differential equations, random networks growth, migration flows, traffic flow, flow in pipelines, flows in supply chain networks

## Abstract

This review paper is devoted to a brief overview of results and models concerning flows in networks and channels of networks. First of all, we conduct a survey of the literature in several areas of research connected to these flows. Then, we mention certain basic mathematical models of flows in networks that are based on differential equations. We give special attention to several models for flows of substances in channels of networks. For stationary cases of these flows, we present probability distributions connected to the substance in the nodes of the channel for two basic models: the model of a channel with many arms modeled by differential equations and the model of a simple channel with flows of substances modeled by difference equations. The probability distributions obtained contain as specific cases any probability distribution of a discrete random variable that takes values of 0,1,…. We also mention applications of the considered models, such as applications for modeling migration flows. Special attention is given to the connection of the theory of stationary flows in channels of networks and the theory of the growth of random networks.

## 1. Short Overview of Selected Areas of Research on Flows in Networks

In this review paper, we briefly discuss several areas of the vast research field devoted to flows in networks and mention selected results from these areas. First, we mention several areas of research with an overview of the literature. Our focus is on the models of flows in networks based on differential and difference equations. We present several such models from different areas connected to the theory of flows in networks. Our special attention is devoted to models of flows of substances in channels of networks. We discuss such models containing differential or difference equations. Here, we focus on stationary flows and on the probability distributions connected to the amount of a substance in nodes that belong to a network channel. Finally, we consider a relation between the stationary flow of a substance in a network channel and the theory of growth of random networks.

In 1962, Ford and Fulkerson [1] wrote an influential book devoted to flows in networks. The book contains many results for maximal flows and for minimal-cost flows. It became very popular due to the possible applications of the discussed topics to transportation problems. Over the years, the number of areas of application of the theory started to grow. Results from the research of Ford and Fulkerson have been used to solve problems connected to computer vision and graph theory [2,3,4,5,6]. In mathematical social dynamics, the ideas of Ford and Fulkerson have been used in studies of conflicts in small groups [7], social structures in networks [8], and social influence [9]. Recently, the methodologies and ideas of the theory of flows in networks have found applications in the planning of networks of charging stations for electric vehicles [10], evacuation planning [11,12,13,14], chemical reaction networks [15], ecology [16], assembly line design [17], etc.

One large area of research on flows in networks is devoted to traffic flows and transportation problems in networks. This includes vehicular traffic on urban roads [18,19,20,21,22,23,24,25,26,27,28,29,30,31,32,33], inclusive traffic jams [34,35], railway networks [36,37,38], and air traffic management [39,40]. Flows in pipeline networks, such as oil and gas flows in their corresponding transportation networks [41,42,43,44,45,46,47,48,49], are also often studied. One interesting topic in this research area is the control of material flows or traffic flows in networks [50,51,52,53,54,55].

The aspects of fluid dynamics and hydrology of flows in networks are another vast area of research. This research includes, for an example, flows in networks of microfluidic channels [56,57], flows in networks of irrigation channels [58], and, especially, optimal operation and control of multireservoir systems [59,60,61,62,63,64,65], debris flows in river networks [66], flows in porous media [67,68,69], calibration of water flows in network models [70,71,72], etc.

Economic and biological aspects of network flows are also often modeled. Supply chains were studied in [73,74,75,76,77,78,79], in addition to the studies of commodity networks for energy management [80] and flows in financial networks [81,82,83,84,85,86]. There have also been studies in the areas of forest management [87], disaster relief operations [88], and other applications [89,90,91,92,93,94,95,96,97,98,99]. Very important from the technical and economic points of view are studies of telecommunication, electrical, and computer networks [100,101,102,103,104,105,106,107]. In the areas of medicine and biology, research on network flows concerns topics such as the blood circulatory system in humans [108]—especially the modeling of arterial flows in networks [109,110]—and metabolic networks [111,112,113,114]. The theory of network flows can also be applied to population and epidemic problems [115,116], especially for problems of human migration. Additional areas of research can be found in [117,118,119,120,121].

The mathematical aspects of network flow modeling are also of primary interest [122,123,124,125,126,127,128,129,130]. There has been special interest in research on the maximum flow problem [131,132,133,134,135] and finding the shortest path [136,137,138]. The algorithmic aspects of flows in networks also attract great attention [139,140,141,142,143,144,145,146,147,148,149]. Especially interesting are the results on algorithms for minimax transport problems [150,151,152,153], algorithms for convex-cost network flows [154,155,156], algorithms for multicommodity networks [157,158,159,160,161,162], e.g., for high-school timetabling [163], parallel algorithms for network flows [164,165,166,167], and distributed computation [168]. There is research on flows in dendritic and looped networks [169]. The Riemann problems [170,171,172,173,174,175] are a very current topic connected to numerical solutions of a system of model equations for network flows. Let us note also that the mathematical parts of these studies are connected to graph theory, combinatorics, and linear programming [176,177,178,179,180].

Below, we will concentrate our attention on a discussion on mathematical models of flows in networks based on differential equations, with an emphasis on models of flows in network channels and distributions of substances at the nodes of the channels for the state of a stationary flow. The kind of flowing substance can be, for example, a resource that flows through the nodes of the channel. The possible applications are, e.g., (I) to cause a substance to flow in a channel in order to be part of an industrial process in some nodes of the channel or (II) the evaluation and prognosis of migration flows of humans and animals. Application (II) is interesting, as probability and deterministic models of human migration [181,182,183,184,185] are often used to make decisions about the economic development of parts of a country [186,187,188,189,190]. In addition, the above models were used for the analysis of processes of migration [191,192,193,194,195,196,197,198,199], for the analysis of ideological struggles [200,201], for the description of waves and distributions in population systems [202,203,204,205,206,207], etc. For an example, in [199], the motion of a substance in a finite channel of a network that split into two arms in a node of the network was discussed. In addition, one more split existed in the secondary arm of the channel. For this configuration of the channel, analytical relationships were obtained for the distribution of the substance in the nodes of the channel for the case of a stationary regime of the motion of the substance. The application of the obtained results was to the motion of migrants in a chain of countries with different probabilities for their obtaining permission to stay in the different countries of the channel. Migrants in a country earn money and send some of it to their home countries. The existence of analytical relationships for migrant distributions allows us to evaluate the amount of this money. An evaluation can be performed for a selected country of the channel. Such evaluations can be performed for several countries of the channel at once.

Another kind of problem was discussed in [201]. There, a model of ideological competition was described. In this model, the migration of populations was taken into account. The model described interactions between global populations of non-believers and followers of different ideologies in the presence of the possibility for conversion from one ideology to another by means of the influence of mass media and interpersonal relationships. In addition, different birth rates could exist for the followers of different ideologies. Competition among ideologies may happen in one or several regions in a space.

Some of the models mentioned below can be connected to urn models. By means of this connection, the discussed models can be applied to a wide range of problems from the area of genetics, through the areas of clinical trials and biology, and to the areas of social dynamics or military theory [208,209,210].

## 2. Models of Network Flows Containing Differential Equations

Differential equations are widely used in models of flows in networks. Below, we mention a small selection of basic models. First of all, we consider several models of vehicular traffic. Modeling vehicular traffic [211,212,213,214,215,216,217,218,219,220,221,222,223,224,225,226,227,228] is an important task as this traffic is connected to large social problems, such as environmental pollution and the congestion of cities. It is important froman economic and social point of view to have rational planning and management of vehicle flows. Such planning and management are not possible only on the basis of simple experimental approaches. Thus, vehicular traffic leads not only to interesting engineering tasks, but also to different mathematical problems. We must mention the microscopic models of vehicular traffic [215,218,226]. In these models, each single vehicle is modeled, and the dynamics are based on a system of ordinary differential equations of the second order. In macroscopic and kinetic models, one uses a continuum hypothesis. The motion of single cars is not of interest for these models. This leads to simple models based on several partial differential equations. These models are appropriate for modeling the motion of a large number of cars, and in this case, we can introduce quantities that are continuous functions of space, such as macroscopic density or kinetic distribution functions.

The basic idea of macroscopic modeling comes from Lighthill, Whitham, and Richards. The idea is that the Euler and Navier–Stokes equations (known from fluid dynamics) may also describe motion of cars along a road. The cars are modeled as small particles, and the density of these particles is the main quantity of the mode [18,19]. The model is based on the equation

(1) $$\frac{\partial \rho (x,t)}{\partial t}+\frac{\partial f[\rho (t,x)]}{\partial x}=0.$$

In (1), ρ(x,t) is the density of cars, *t* is the time, and *x* is the position. f(ρ)=ρv(ρ) denotes the flux of the cars. v(ρ) depends only on ρ. This density can be in the interval $\rho \in [0,\rho_{max}]$ [20,229]. Models based on Equation (1) are called first-order macroscopic models.

Roads form junctions. A junction is a point where we have $n_{1}$ incoming roads and $n_{2}$ outgoing roads. The incoming roads are described by the interval (−∞,0), and the outgoing roads are described by the interval (0,+∞). Thus, the flow through a junction is modeled by $n_{1}+n_{2}$ differential equations:

(2) $$\frac{\partial \rho_{i}\left(x,t\right)}{\partial t}+\frac{\partial f[\rho_{i}\left(t,x\right)]}{\partial x}=0;\;\;\;x\in \left(-\infty ,0\right);\;\;\;i\in \left\{1,\ldots ,n_{1}\right\},$$

for the case of incoming roads, and

(3) $$\frac{\partial \rho_{i}\left(x,t\right)}{\partial t}+\frac{\partial f[\rho_{i}\left(t,x\right)]}{\partial x}=0;\;\;\;x\in \left(0,\infty \right);\;\;\;i\in \left\{n_{1}+1,\ldots ,n_{1}+n_{2}\right\},$$

for the case of outgoing roads. The junction is at x=0, and the preservation of the number of cars is assumed:

(4) $$\sum_{i=1}^{n_{1}}f\left[\rho_{i}\left(t,0\right)\right]=\sum_{i=n_{1}+1}^{n_{1}+n_{2}}f\left[\rho_{i}\left(t,0\right)\right].$$

Additional conditions have to be applied to obtain a unique solution of (2)–(4). One often-used condition is connected to the preferences of drivers. The preferences $p_{kl}\in \left[0,1\right]$ are the percentages of traffic that come from the incoming road *l* and go to the outgoing road *k*. They form the matrix $P=\{P_{kl}\}$ with elements $P_{kl}=p_{n_{1}+k,l}$, $k=1,\ldots ,n_{2}$; $l=1,\ldots ,n_{1}$. This matrix connects the vectors of fluxes:

(5) $${\left(\phi_{n_{1}+1},\ldots ,\phi_{n_{1}+n_{2}}\right)}^{T}=A\cdot {\left(\phi_{1},\ldots ,\phi_{n_{1}}\right)}^{T},$$

where $\phi_{i}$ is the flux entering the *i*-th incoming road or the flux that enters the *i*-th outgoing road. The additional condition is the condition for flux maximization through the junction

$$\max(\phi_{1}+\cdots +\phi_{n_{1}}).$$

Thus, the solution of the Riemann problem for the junction is unique, and the Cauchy problem has a solution that is entropy-admissible for every time interval [0,T] [229].

One can use a more complicated equation than (1). One possibility is to add a diffusion term [230,231]:

(6) $$\frac{\partial \rho (x,t)}{\partial t}+\frac{\partial f[\rho (t,x)]}{\partial x}+\alpha \frac{\partial [\kappa (\rho )\partial \rho /\partial x]}{\partial x}=0.$$

In (6), the parameter α>0 accounts for the strength of the diffusion. The nonlinear diffusion coefficient is denoted as κ(ρ). For the specific case when κ(ρ)=κ>0, Equation (6) coincides with the linear heat equation with added nonlinear advection.

If the traffic on the network intensifies, one has to use models containing nonlinearities with respect to ρ. One such model is the famous Aw–Rascle– Zhang model [232,233,234,235,236,237,238,239]. The model is based on two equations:

(7) $$\begin{array}{c} \frac{\partial \rho }{\partial t}+\frac{\partial (\rho v)}{\partial x}=0,\;\;\;\frac{\partial [v+p(\rho )]}{\partial t}+v\frac{\partial [v+p(\rho )]}{\partial x}=0. \end{array}$$

ρ(x,t) is the density of the flow and v(x,t) is the velocity of the flow. p(ρ) has to be a smoothly increasing function. Aw and Rascle chose it as follows:

(8) $$p\left(\rho \right)=\rho^{\gamma },\;\;\;\gamma >0.$$

Thus, the system of the model equation for a junction that has $n_{1}$ incoming roads and possesses $n_{2}$ outcoming roads can be written as

(9) $$\begin{array}{c} \frac{\partial \rho }{\partial t}+\frac{\partial (y-\rho^{\gamma +1})}{\partial x}=0,\;\;\;\frac{\partial y}{\partial t}+\frac{\partial (y^{2}/\rho -y\rho^{\gamma })}{\partial x}=0. \end{array}$$

where $y=\rho v+\rho^{\gamma +1}$ is the generalized momentum. The system of the model equation for the junction is as follows. For the incoming roads (x<0, $i=1,\ldots ,n_{1}$),

(10) $$\begin{array}{c} \frac{\partial \rho_{i}}{\partial t}+\frac{\partial (y_{i}-\rho_{i}^{\gamma +1})}{\partial x}=0,\;\;\;\frac{\partial y_{i}}{\partial t}+\frac{\partial (y_{i}^{2}/\rho_{i}-y_{i}\rho_{i}^{\gamma })}{\partial x}=0. \end{array}$$

For the outgoing roads (x>0, $i=n_{1}+1,\ldots ,n_{1}+n_{2}$), we have again the system of Equation (10). In order to have a unique solution of this model, one has to require as additional conditions the maximization of the velocity of the cars, the minimization of the density, and the minimization of the full variation of the solution [239]. Other sets of additional conditions are also possible [240]. Different kinds of traffic models, such as a phase transition model, can also be considered. For several examples, see [241,242,243,244,245,246,247].

Another class of flows in networks that are modeled by differential equations are data flows in communication networks [100,248,249,250,251]. In this class of models, the nodes represent the servers and the edges represent the connections between the nodes. Transmission of information packets happens between the nodes. This transmission is modeled by the equation

(11) $$\frac{\partial \rho }{\partial t}+\frac{\partial g(\rho )}{\partial x}=0.$$

The density of the packets above is denoted by ρ, and g(ρ) is connected to the constant velocity of the packets *v* and to the maximum density $\rho_{max}$ of the packets as follows:

(12) $$\begin{array}{ccc} g(\rho ) & = & v\rho ,\;\;\;0\leq \rho \leq \mu ;\\ g(\rho ) & = & v\mu \frac{\rho_{max}-\rho }{\rho_{max}-\mu },\;\;\;\mu \leq \rho \leq \rho_{max}. \end{array}$$

The parameter $\mu \in (0,\rho_{max})$ accounts for the probability of packet loss. In order to complete the model for a single transmission line, an equation for a traffic-type function π(x,t) should be added [248]:

(13) $$\frac{\partial \pi }{\partial t}+v\left(\rho \right)\frac{\partial \pi }{\partial x}=0.$$

The model can be extended for the situation in which the junctions have $n_{1}$ incoming lines and $n_{2}$ outgoing lines. A unique solution at a nodal point can be obtained with additional conditions on the flux passing through the junction. This flux should have the maximum possible value. After the determination of the flux through the junction, the flux is distributed among the transmission lines by means of appropriate priority coefficients.

Our next example is connected to models of flows in open channels [252,253,254]. The model equations are the Saint Venan equations:

(14) $$\begin{array}{c} \frac{\partial a}{\partial t}+\frac{\partial (va)}{\partial x}=0,\;\;\;\frac{\partial v}{\partial t}+\frac{\partial (V^{2}/2+gH\left(a\right))}{\partial x}+S\left(a,v\right)=0. \end{array}$$

In (14), *g* is the acceleration of gravity, *v* denotes the velocity, and *a* denotes the wet cross-section with the corresponding height of the water H(a). S(a,v) accounts for the slope of the channel and for the friction because of the slope. Equation (14) describes a single channel. For the case of a node that is a junction among *m* channels, we need *m* equations of the same kind as (14) with additional conditions at the node [252,255]:

(15) $$\begin{array}{c} \sum_{i=1}^{m}a_{i}v_{i}=0,\;\;\;\frac{v_{i}^{2}}{2}+gh\left(a_{i}\right)=\frac{v_{1}^{2}}{2}-gh\left(a_{1}\right),i=1,\ldots ,m. \end{array}$$

Our next example is connected to supply chain networks [256,257,258]. Let us consider a network of supply chains that connect *m* suppliers. We consider a single node in this network. *l* is the number of incoming suppliers and m−l is the number of outgoing suppliers. The structure of an outgoing supplier consists of a processor, and there is a queue in front of it. Only a processor forms the incoming supplier. The model for the work done by a processor is

(16) $$\frac{\partial \rho_{i}\left(x,t\right)}{\partial t}+\frac{\partial v_{i}\rho_{i}\left(x,t\right)}{\partial x}=0.$$

where i=1,…,n, and *x* and *t* are nonnegative real numbers. $v_{i}$ is the constant processing velocity. The density of goods in the *i*-th processor is denoted by $\rho_{i}$. $v_{i}<0$ for i=1,…,l. $v_{i}>0$ for i=l+1,…,m. There, the conservation of mass for the load $q_{k}$ of goods (which are stored in the k-th queue) is

(17) $$\frac{dq}{dt}=f_{k}^{in}\left[u\left(0,t\right),t\right]-f_{k}^{out}\left(q_{k}\right),\;\;\;k+l+1,\ldots ,m.$$

In (17), $f_{k}^{in}$ and $f_{k}^{out}$ denote the inflow and the outflow from the queue attached to the *k*-th processor. This queue is fueled as follows:

(18) $$f_{k}^{in}\left[\rho \left(0,t\right),t\right]=\sum_{j=1}^{l}a_{jk}\left(t\right)v_{j}\rho_{j}\left(0,t\right),\;\;\;k+l+1,\ldots ,m.$$

In (18), $a_{jk}\left(t\right)$, j=1,…,l, k=l+1,…,m are elements of a distribution matrix and represent the percentages of goods going out of the processor *j* and coming to the queue in the quantity *k*. The outflow is modeled as

(19) $$f_{k}^{out}\left(q\right)=\min\left(\frac{q_{k}\left(t\right)}{\nu },\mu_{k}\right),\;\;\;k=l+1,\ldots ,m,$$

where ν>0 is a (small) relaxation parameter. $\mu_{k}$ is the maximum capacity of the k-th processor.

Finally, we present an example of a model of flow of gas in a pipeline network [45]. We write the variant of the model for the case of a horizontal pipeline network. In this case, the model equations for the gas flow are:

(20) $$\begin{array}{c} -\frac{\partial p}{\partial x}=\frac{\partial (\rho w)}{\partial t}+\frac{\lambda }{2D}\rho w^{2}+\frac{\partial {\left(\rho w\right)}^{2}}{\partial x};\;\;\;-\frac{\partial p}{\partial t}=c^{2}\frac{\partial (\rho w)}{\partial x}. \end{array}$$

In (20), *c* is the speed of sound in the gas. The gas velocity averaged over the cross-sectional area of the pipeline is denoted by w(x,t). ρ(x,t) is the density of the gas averaged over the cross-sectional area of the pipeline; λ is the friction coefficient for the fluid in the pipeline; *D* is the pipeline diameter; p(x,t) is the gas pressure averaged over the cross-sectional area of the pipeline.

The node pressures in the pipeline network are computed by the equations

(21) $$\begin{array}{c} \frac{dm_{j}}{dt}=\sum_{i=1}^{n}B_{ij}Q_{m(ij)}-Q_{m(j)}. \end{array}$$

where *n* in (21) is the number of pipes connected to the node. *m* is the mass of the gas in the node; $Q_{m(j)}$ is the mass flow of the gas in the *j*-th node; $Q_{m(ij)}$ is the flow into or out of the *j*-th node in the *i*-th pipe connected to the *j*-th node; $B_{ij}=1$ if the flow $Q_{m(ij)}$ goes into the *j*-th node; $B_{ij}=-1$ if the flow $Q_{m(ij)}$ goes out of the *j*-th node.

The list of the differential equation models for flows in networks can be continued. A nice overview of such models can be found in [256]. Additional models can be seen in [231,259,260,261,262,263,264].

Models of flows in networks can contain numerous differential equations. As a consequence, one often has to solve these equations numerically [265,266,267,268,269,270,271,272,273,274,275,276]. There are cases in which the corresponding model equations are not large in number, or we are interested in the situation in just one or several nodes of the network. In such cases, one can search for exact solutions of the model equation(s). One powerful methodology for obtaining exact solutions for the case of nonlinear model differential equations is the SEsM (Simple Equations Method) [277,278,279,280,281,282,283,284,285,286,287,288,289,290,291]. We have only mentioned this methodology here, as it will be discussed in detail in other feature paper of this special issue.

## 3. Differential and Difference Equations for Modeling Flows in Channels of Networks: Selected Results

The focus below is on models of flows in network channels that are based on differential equations (in the case of continuous time) and difference equations (in the case of discrete time).

### 3.1. Flow of a Substance in a Channel Constructed from Arms with an Infinite Number of Nodes Each

**Figure 1:**
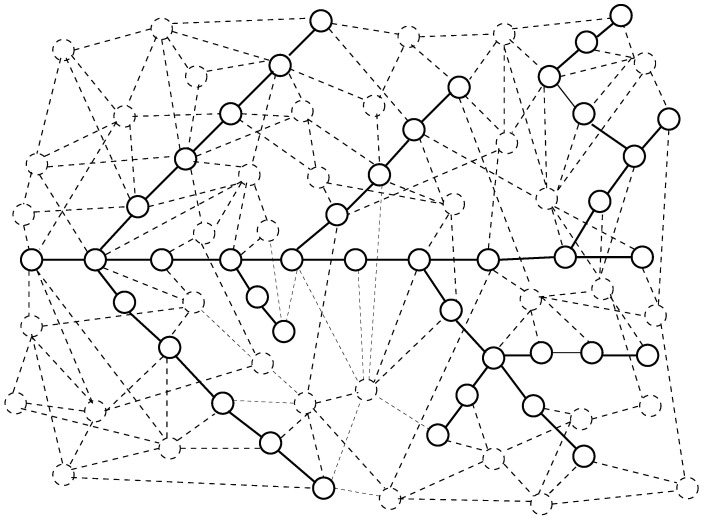
The network and the channel. Solid lines denote nodes and edges that belong to the channel. Dashed lines denote the other nodes and edges of the network.

This is an example of a model based on differential equations. We consider the motion of a substance in a network channel that contains many arms. The model of the motion of a substance through the channel is an extension of the model from [195,206]. The channel consists of chains of nodes of a network; see Figure 1.

**Figure 2:**
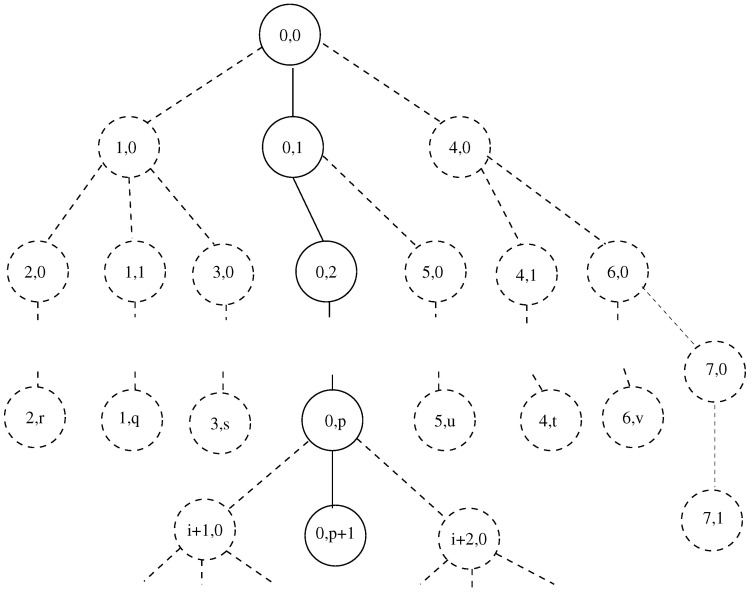
Numbering of the nodes of the channel. The lower two indexes of the numbers of the nodes are shown.

The numbering of the nodes of the channel is shown in Figure 2. The notation $N_{i,j}^{a,b}$ is used for a node of the channel. The position of a node in the current arm is specified by the lower indexes. *i* is the number of the arm of the channel. *j* denotes the number of nodes of the *i*-th arm. The upper indexes denote the origin of arm *i*. *a* is number of the parent arm from which arm *i* splits. *b* is the number of the node of arm *a* at which this split happens. $N_{4,6}^{2,3}$ indicates the sixth node of arm 4, which splits at node 3 of arm 2.

It is assumed that a substance can enter the channel from the environment. This happens only through the 0-th node of the main arm of the channel. This arm has a label of q=0. The motion of the substance is only in one direction: from nodes with smaller values of index *j* to nodes with larger values of index *j*. Most nodes in an arm of the channel are connected to only two neighboring nodes. There are special nodes. There, a split of an arm happens. These nodes can be connected to additional nodes. The substance can leak from the channel. It can leave the nodes of channel and move into the environment.

The first node at which arm *i* begins is denoted as the 0-th node of the *i*-th arm. The 0-th node is the first one after the splitting of arm *a* at node *b*. The 0-th node of arm 0 is the “environment” from which a substance flows towards the 0-th node of arm 1.

Let us have an arm *q* that splits at node ($a_{q},b_{q}$) of the network. An amount $x_{q}^{a_{q},b_{q}}$ of a substance is present in the nodes of this arm. We denote as $x_{q,i}^{a_{q},b_{q}}$ the amount of the substance that is in the *i*-th node of the *q*-th arm of the channel. Then, $x_{q}^{a_{q},b_{q}}=\sum_{i=0}^{\infty }x_{q,i}^{a_{q},b_{q}}.$ The fractions $y_{q,i}^{a_{q},b_{q}}=x_{q,i}^{a_{q},b_{q}}/x_{q}^{a_{q},b_{q}}$ can be thought of as values of the probability distribution of a discrete random variable $\zeta_{q}$. They describe the probability $y_{q,i}^{a_{q},b_{q}}=p_{q}^{a_{q},b_{q}}\left(\zeta_{q}=i\right),\;i=0,1,\ldots$. $p_{q}^{a_{q},b_{q}}$ is the probability that the random variable has the value *i*. The full amount of the substance in the studied channel is $x=\sum_{q=0}^{M}x_{q}^{a_{q},b_{q}}=\sum_{q=0}^{M}\sum_{i=0}^{\infty }x_{q,i}^{a_{q},b_{q}}.$ Here, M+1 is the number of arms of the channel. Then, the distribution of the substance in the channel is $z_{q,i}^{a_{q},b_{q}}=\frac{x_{q,i}^{a_{q},b_{q}}}{x}$.

The amount $x_{q,i}^{a_{q},b_{q}}$ of the substance can change. For example, the amount $s_{q}^{a,b}$ of the substance can enter arm *q* from the external environment through the 0-th cell of the arm. For the arm where q=0, the substance $s_{0}^{0,0}$ enters the arm from the environment. For the arms with numbers q≠0, the substance in the amount of $s_{q}^{a,b}$ is part of the substance from node (a,b) of the parent arm.

Another process occurs when a transfer of a substance between the nodes of the channel is possible. Then, the amount $f_{q,i}^{a,b}$ from $x_{q,i}^{a,b}$ moves from the *i*-th node to the i+1-th node of the *q*-th arm. The amount $g_{q,i}^{a,b}$ of $x_{q,i}^{a,b}$ leaks out of the *i*-th node of the *q*-th arm into environment of the arm of the channel. Two kinds of leakage are taken into account. The first kind is leakage into the environment. This leads to loss of the substance for the channel. Another kind of leakage is leakage into other arms of the channel, which begins from the node *b* of arm *a*. This leakage is connected to the substance $s_{q}^{a,b}$, which enters corresponding child arm of the channel that splits from node *b* of arm *a*.

The process of motion of the substance is assumed to be continuous in time. The motion of the substance among the nodes of the *q*-th arm is modeled mathematically with a system of ordinary differential equations:

(22) $$\begin{array}{ccc} \frac{dx_{q,0}^{a,b}}{dt} & = & s_{q}^{a,b}-f_{q,0}^{a,b}-g_{q,0}^{a,b};\\ \frac{dx_{i,q}^{a,b}}{dt} & = & f_{q,i-1}^{a,b}-f_{q,i}^{a,b}-g_{q,i}^{a,b},\;i=1,2,\ldots . \end{array}$$

We discuss the stationary regime of the flow of the substance. For this regime, $dx_{i,q}^{a,b}/dt=0$, i=0,1,…. The corresponding quantities are marked with ${}^{*}$. The following forms of amounts of substances are assumed in (22) ($\alpha_{i},\beta_{i},\gamma_{i},\sigma_{0}$ are parameters): $s_{0}^{0,0}=\sigma_{0}x_{0,0}^{0,0}>0$; $s_{q}^{a,b}=\delta_{q}x_{a,b}^{c,d}$; $1\geq \delta_{q}\geq 0$; $f_{q,i}^{a,b}=\left(\alpha_{q,i}^{a,b}+i\beta_{q,i}^{a,b}\right)x_{q,i}^{a,b}$; $1>\alpha_{q,i}^{a,b}>0$, $1\geq \beta_{q,i}^{a,b}\geq 0$; $g_{q,i}^{a,b}=\gamma_{q,i}^{*a,b}x_{q,i}^{a,b}$; $1\geq \gamma_{q,i}^{*a,b}\geq 0.$ Indexes *c* and *d* describe the parent arm and the parent node of arm *c* for arm *q*. $\beta_{q,i}^{a,b}$ accounts for the conditions that help the substance to leave node *i* more quickly. $\gamma_{q,i}^{*a,b}=\gamma_{q,i}^{a,b}+\sum_{p\in (q,i)}\delta_{p,q,i}^{a,b}$ accounts for the leakages in cells. $\gamma_{q,i}^{a,b}$ is the leakage from the *i*-th node of the *q*-th arm into the environment. $\delta_{p,q,i}^{a,b}$ denotes the leakage into the nodes splitting from the *i*-th node of the *q*-th arm. The notation p∈(q,i) denotes all arms that arise from node *i* of arm *q*.

The model system of Equation (22) for the *q*-th arm of the channel is

(23) $$\begin{array}{ccc} \frac{dx_{q,0}^{a,b}}{dt} & = & s_{q}^{a,b}-\alpha_{q,0}^{a,b}x_{q,0}^{a,b}-\gamma_{q,0}^{*a,b}x_{q,0}^{a,b};\\ \frac{dx_{q,i}^{a,b}}{dt} & = & \left[\alpha_{q,i-1}^{a,b}+\left(i-1\right)\beta_{q,i-1}^{a,b}\right]x_{q,i-1}^{a,b}-\left(\alpha_{q,i}^{a,b}+i\beta_{q,i}^{a,b}+\gamma_{q,i}^{*a,b}\right)x_{q,i}^{a,b};\;\;i=1,2,\ldots \end{array}$$

The solution of (23) with increasing time tends to the solution for the stationary case. Then,

(24) $$x_{q,0}^{*a,b}=\frac{s_{q}^{a,b}}{\alpha_{0,q}^{a,b}+\gamma_{0,q}^{*,a,b}}\;\;,x_{r,0}^{*q,m}=\frac{\delta_{r,q,m}^{a,b}}{\alpha_{r,0}^{q,m}+\gamma_{r,0}^{q,m}}x_{q,m}^{*c,d},\;\;x_{q,i}^{*a,b}=\frac{\alpha_{q,i-1}^{a,b}+\left(i-1\right)\beta_{q,i-1}^{a,b}}{\alpha_{q,i}^{a,b}+i\beta_{q,i}^{a,b}+\gamma_{q,i}^{*a,b}}x_{q,i-1}^{*a,b},\;i=1,2,\ldots .$$

Equation (24) leads to (i=1,2,…):

(25) $$x_{q,i}^{*a,b}=\frac{\prod_{j=0}^{i-1}\left[\alpha_{q,i-j-1}^{a,b}+\left(i-j-1\right)\beta_{q,i-j-1}^{a,b}\right]}{\prod_{j=0}^{i-1}\left[\alpha_{q,i-j}^{a,b}+\left(i-j\right)\beta_{q,i-j}^{a,b}+\gamma_{q,i-j}^{*a,b}\right]}x_{q,0}^{*a,b}$$

The amount of the substance in all of the nodes of arm *q* is $x_{q}^{*a,b}=\sum_{i=0}^{\infty }x_{q,i}^{*a,b}=x_{q,0}^{*a,b}+\sum_{i=1}^{\infty }\frac{\prod_{j=0}^{i-1}\left[\alpha_{q,i-j-1}^{a,b}+\left(i-j-1\right)\beta_{q,i-j-1}^{a,b}\right]}{\prod_{j=0}^{i-1}\left[\alpha_{q,i-j}^{a,b}+\left(i-j\right)\beta_{q,i-j}^{a,b}+\gamma_{q,i-j}^{*a,b}\right]}x_{q,0}^{*a,b}.$ Then, the probability distribution $y_{q,i}^{*a,b}=x_{q,i}^{*a,b}/x_{q}^{*a,b}$ is

(26) $$\begin{array}{c} y_{q,0}^{*a,b}=\frac{1}{1+\sum_{i=1}^{\infty }\frac{\prod_{j=0}^{i-1}\left[\alpha_{q,i-j-1}^{a,b}+\left(i-j-1\right)\beta_{q,i-j-1}^{a,b}\right]}{\prod_{j=0}^{i-1}\left[\alpha_{q,i-j}^{a,b}+\left(i-j\right)\beta_{q,i-j}^{a,b}+\gamma_{q,i-j}^{*a,b}\right]}};\;\;\;y_{q,i}^{*a,b}=\frac{\frac{\prod_{j=0}^{i-1}\left[\alpha_{q,i-j-1}^{a,b}+\left(i-j-1\right)\beta_{q,i-j-1}^{a,b}\right]}{\prod_{j=0}^{i-1}\left[\alpha_{q,i-j}^{a,b}+\left(i-j\right)\beta_{q,i-j}^{a,b}+\gamma_{q,i-j}^{*a,b}\right]}}{1+\sum_{i=1}^{\infty }\frac{\prod_{j=0}^{i-1}\left[\alpha_{q,i-j-1}^{a,b}+\left(i-j-1\right)\beta_{q,i-j-1}^{a,b}\right]}{\prod_{j=0}^{i-1}\left[\alpha_{q,i-j}^{a,b}+\left(i-j\right)\beta_{q,i-j}^{a,b}+\gamma_{q,i-j}^{*a,b}\right]}}\\ i=1,2,\ldots \end{array}$$

The probability distribution corresponding to the distribution of a substance in a channel containing *M* arms (M=1,2,…) can also be written. The amount of the substance in the arms of the channel reads $x^{*}=\sum_{q=0}^{M}x_{q,0}^{*a,b}\left[1+\sum_{i=1}^{\infty }\frac{\prod_{j=0}^{i-1}\left[\alpha_{q,i-j-1}^{a,b}+\left(i-j-1\right)\beta_{q,i-j-1}^{a,b}\right]}{\prod_{j=0}^{i-1}\left[\alpha_{q,i-j}^{a,b}+\left(i-j\right)\beta_{q,i-j}^{a,b}+\gamma_{q,i-j}^{*a,b}\right]}\right].$ Then, for the 0-th node of the *p*-th arm of the channel, as well as for the *i*-th node of the *p*-th arm of the channel, we have (i=1,2,…):

(27) $$\begin{array}{c} z_{p,0}^{*a_{p},b_{p}}=\frac{x_{p,0}^{*,a_{p},b_{p}}}{\sum_{q=0}^{M}x_{q,0}^{*a,b}\left[1+\sum_{i=1}^{\infty }\frac{\prod_{j=0}^{i-1}\left[\alpha_{q,i-j-1}^{a,b}+\left(i-j-1\right)\beta_{q,i-j-1}^{a,b}\right]}{\prod_{j=0}^{i-1}\left[\alpha_{q,i-j}^{a,b}+\left(i-j\right)\beta_{q,i-j}^{a,b}+\gamma_{q,i-j}^{*a,b}\right]}\right]}, \end{array}$$

(28) $$\begin{array}{c} z_{p,i}^{*a_{p},b_{p}}=\frac{\frac{\prod_{j=0}^{i-1}\left[\alpha_{p,i-j-1}^{a,b}+\left(i-j-1\right)\beta_{p,i-j-1}^{a,b}\right]}{\prod_{j=0}^{i-1}\left[\alpha_{p,i-j}^{a,b}+\left(i-j\right)\beta_{p,i-j}^{a,b}+\gamma_{p,i-j}^{*a,b}\right]}x_{p,0}^{*a,b}}{\sum_{q=0}^{M}x_{q,0}^{*a,b}\left[1+\sum_{i=1}^{\infty }\frac{\prod_{j=0}^{i-1}\left[\alpha_{q,i-j-1}^{a,b}+\left(i-j-1\right)\beta_{q,i-j-1}^{a,b}\right]}{\prod_{j=0}^{i-1}\left[\alpha_{q,i-j}^{a,b}+\left(i-j\right)\beta_{q,i-j}^{a,b}+\gamma_{q,i-j}^{*a,b}\right]}\right]} \end{array}$$

If the channel contains only one arm, the probability distributions written above reduce to the distribution discussed in [195]. This distribution is related to the distribution of Waring, which is a long-tailed distribution.

The model discussed here leads to many possible kinds of probability distributions. The most simple of them are for a channel that has a single arm. Such distributions are described in [183,191,192,195]. They can be connected to the Zipf distribution, Waring distribution, binomial distribution, Yule–Simon distribution, etc.

This model has many applications. It can be applied to the motion of a substance in technological systems or for the study of the motion of goods in logistic networks. This model can also be applied for the study of the motion of migrants in channels of human migration [292].

### 3.2. Model of a Flow in a Channel of a Network Based on Difference Equations

**Figure 3:**
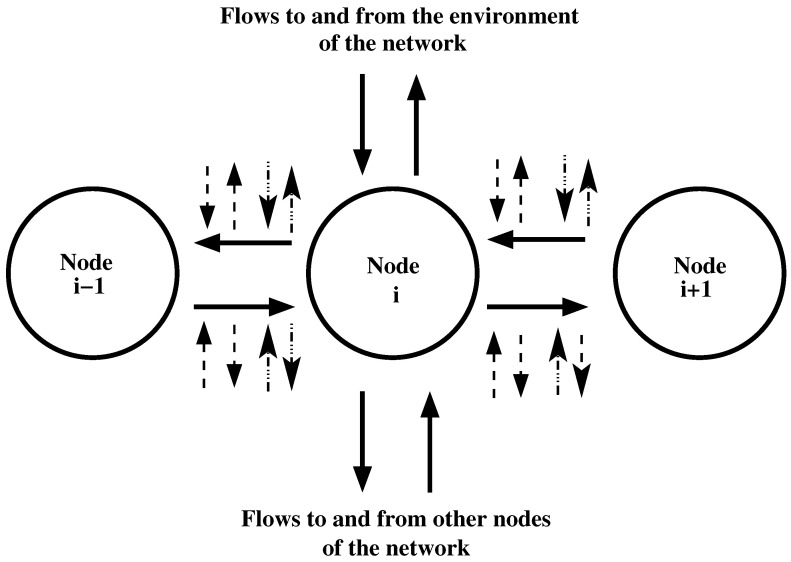
Flows connected with the *i*-th node of the channel. Nodes 0 and *N* exchange substances with only one of the other nodes of the channel. There is a possibility for an exchange of substances among flows between the nodes and (i) the network (arrows with dashed lines) or (ii) the environment of the network (arrows with dot-dashed lines).

Difference equations are used to model flows of a substance in channels of networks for the case of discrete time. A basic model is as follows [293]. We consider a channel of a network that is a chain of N+1 nodes (labeled from 0 to *N*) connected by edges. Each edge connects two of the nodes, and each of the nodes is connected to two edges, with the exception of two nodes. These nodes are the 0-th node and the *N*-th node. They are connected to one edge. A substance can move along the channel. In addition to motion, there are processes of exchange of substances between the channel, the environment of the network, and the rest of the network; see Figure 3. The *i*-th node exchanges substance with nodes that have numbers (i−1) and (i+1). Node *i* can also obtain and send a substance to other nodes of the network that are outside the channel. The same can happen between node *i* and the environment of the network. There can be a motion of a substance from a channel node to a node of the network. This process is called leakage. Leakage is also the motion of a substance from a node of the channel toward the environment of the network. There can be also inflow of a substance; this is the motion of the substance from a network node that does not belong to the channel to a node of the channel. Inflow is also the motion of the substance from the environment of the network toward a node of the channel. There can be additional exchanges of substances connected to the flows between the nodes. These exchanges are denoted in Figure 3 by arrows with dashed or dot-dashed lines.

The time in the model is divided into time intervals. At each time interval, the substance in a node may participate in several processes. First of all, the substance may remain in the same node. Then, the substance may move from node *m* to node m+1 or from node *m* to node m−1. Finally, the substance may leak from node *m*. There is a possibility for a leakage from node *m* to the nodes of the network. Another possibility is a leakage from node *m* to the environment of the network. An inflow of the substance into a node is also possible. The inflow can be of two kinds. The first kind is an inflow from other nodes of the network into node *m* of the channel. The second kind of inflow is from the environment of the network into the channel node *m*.

Additional kinds of processes are possible for flows between nodes of a channel. First of all, a substance may move from the flow to a node of the network that is external to the channel (in Figure 3, such flows are denoted by arrows with a dashed line pointing from the flow). Then, the substance may move from a node of the network that is external to the channel to the flow between two nodes of the channel (in Figure 3, such flows are denoted by arrows with a dashed line pointing to the flow). As a third possibility, the substance may move from the flow to the network environment (in Figure 3, such flows are denoted by arrows with a dot-dashed line pointing from the flow). Finally, the substance may move from the network environment to the flow between two nodes of the channel (in Figure 3, such flows are denoted by a dot-dashed line pointing to the flow).

Next, we write the equations of the model. The discrete time is denoted as $t_{k}$, k=0,1,2,…. $x_{i}\left(t_{k}\right)$ is the amount of the substance in the *i*-th node of the channel at the occurrence of the time interval $\left[t_{k},t_{k}+\Delta t\right]$. The model equations are [293]:

(29) $$\begin{array}{ccc} x_{0}\left(t_{k+1}\right) & = & x_{0}\left(t_{k}\right)+i_{0}^{e}\left(t_{k}\right)-o_{0}^{e}\left(t_{k}\right)-o_{0}^{c}\left(t_{k}\right)+i_{0}^{c}\left(t_{k}\right)-o_{0}^{n}\left(t_{k}\right)+i_{0}^{n}\left(t_{k}\right)-h_{0}^{e}\left(t_{k}\right)-\\ & & h_{0}^{n}\left(t_{k}\right)+p_{0}^{e}\left(t_{k}\right)+p_{0}^{n}\left(t_{k}\right),\\ x_{i}\left(t_{k+1}\right) & = & x_{i}\left(t_{k}\right)+i_{i}^{e}\left(t_{k}\right)-o_{i}^{e}\left(t_{k}\right)+o_{i-1}^{c}\left(t_{k}\right)-i_{i-1}^{c}\left(t_{k}\right)-o_{i}^{c}\left(t_{k}\right)+i_{i}^{c}\left(t_{k}\right)-o_{i}^{n}\left(t_{k}\right)+i_{i}^{n}\left(t_{k}\right)-\\ & & h_{i}^{e}\left(t_{k}\right)-h_{i}^{n}\left(t_{k}\right)+p_{i}^{e}\left(t_{k}\right)+p_{i}^{n}\left(t_{k}\right)-f_{i-1}^{e}\left(t_{k}\right)-f_{i-1}^{n}\left(t_{k}\right)+g_{i-1}^{e}\left(t_{k}\right)+g_{i-1}^{n}\left(t_{k}\right),\\ & & i=1,\ldots ,N-1\\ x_{N}\left(t_{k+1}\right) & = & x_{N}\left(t_{k}\right)+i_{N}^{e}\left(t_{k}\right)-o_{N}^{e}\left(t_{k}\right)+o_{N-1}^{c}\left(t_{k}\right)-i_{N-1}^{c}\left(t_{k}\right)-o_{N}^{n}\left(t_{k}\right)+i_{N}^{n}\left(t_{k}\right)-f_{N-1}^{e}\left(t_{k}\right)-\\ & & f_{N-1}^{n}\left(t_{k}\right)+g_{N-1}^{e}\left(t_{k}\right)+g_{N-1}^{n}\left(t_{k}\right). \end{array}$$

Above, $i_{n}^{e}\left(t_{k}\right)$ and $o_{n}^{e}\left(t_{k}\right)$ denote the amounts of inflow and outflow of the substance from the environment to the *n*-th channel node. The upper index *e* marks the fact that the quantities are in the environment. Next, the amount of outflow of the substance from the *n*-th node to the (n+1)-th node is denoted as $o_{n}^{c}\left(t_{k}\right)$. The upper index *c* marks the fact that the quantity is for the channel. $i_{n}^{c}\left(t_{k}\right)$ denotes the amount of inflow of the substance from the (n+1)-th node to the *n*-th node. $o_{n}^{n}\left(t_{k}\right)$ and $i_{n}^{n}\left(t_{k}\right)$ denote the amounts of outflow and inflow of the substance between the *n*-th node and the network. The upper index *n* marks the fact that the quantities are for the network. $f_{n}^{e}\left(t_{k}\right)$ denotes the amount of the outflow of the substance from the flow between the *n*-th and n+1-th nodes to the environment of the network. $f_{n}^{n}\left(t_{k}\right)$ denotes the amount of outflow of the substance from the flow between the *n*-th and n+1-th nodes to the network. $g_{n}^{e}\left(t_{k}\right)$ denotes the amount of the inflow of the substance to the flow between the *n*-th and n+1-th nodes from the environment of the network. $g_{n}^{n}\left(t_{k}\right)$ denotes the amount of the inflow of the substance to the flow between the *n*-th and n+1-th nodes from the network. $h_{n}^{e}\left(t_{k}\right)$ denotes the amount of the outflow of the substance from the flow between the n+1-th and *n*-th nodes to the environment of the network. $h_{n}^{n}\left(t_{k}\right)$ denotes the amount of the outflow of the substance from the flow between the n+1-th and *n*-th nodes to the network. $p_{n}^{e}\left(t_{k}\right)$ denotes the amount of the inflow of the substance to the flow between the n+1-th and *n*-th nodes from the environment of the network. $p_{n}^{n}\left(t_{k}\right)$ denotes the amount of the inflow of the substance to the flow between the n+1-th and *n*-th nodes from the network.

Equation (29) presents a general model of the motion of a substance in a chain of nodes of a network. As an example, we present results for a specific case of (29): Linear relationships connect the exchanges between the nodes of the channel, and the amounts of substance in the nodes are given by linear relationships. These relationships are:

(30) $$\begin{array}{ccc} i_{0}^{e}\left(t_{k}\right) & = & \sigma_{0}\left(t_{k}\right)x_{0}\left(t_{k}\right);\;\;o_{0}^{e}\left(t_{k}\right)=\mu_{0}\left(t_{k}\right)x_{0}\left(t_{k}\right)\\ i_{i}^{e}\left(t_{k}\right) & = & \sigma_{i}\left(t_{k}\right)x_{i}\left(t_{k}\right);\;\;o_{i}^{e}\left(t_{k}\right)=\mu_{i}\left(t_{k}\right)x_{i}\left(t_{k}\right),\;\;i=1,\ldots ,N-1\\ i_{N}^{e}\left(t_{k}\right) & = & \sigma_{N}\left(t_{k}\right)x_{N}\left(t_{k}\right);\;\;o_{N}^{e}\left(t_{k}\right)=\mu_{N}\left(t_{k}\right)x_{N}\left(t_{k}\right)\\ i_{0}^{n}\left(t_{k}\right) & = & \epsilon_{0}\left(t_{k}\right)x_{0}\left(t_{k}\right);\;\;o_{0}^{n}\left(t_{k}\right)=\gamma_{0}\left(t_{k}\right)x_{0}\left(t_{k}\right)\\ i_{i}^{n}\left(t_{k}\right) & = & \epsilon_{i}\left(t_{k}\right)x_{i}\left(t_{k}\right);\;\;o_{i}^{n}\left(t_{k}\right)=\gamma_{i}\left(t_{k}\right)x_{i}\left(t_{k}\right),\;\;i=1,\ldots ,N-1\\ i_{N}^{n}\left(t_{k}\right) & = & \epsilon_{N}\left(t_{k}\right)x_{N}\left(t_{k}\right);\;\;o_{N}^{n}\left(t_{k}\right)=\gamma_{N}\left(t_{k}\right)x_{N}\left(t_{k}\right)\\ o_{0}^{c}\left(t_{k}\right) & = & \varphi_{0}\left(t_{k}\right)x_{0}\left(t_{k}\right);\;i_{0}^{c}\left(t_{k}\right)=\delta_{1}\left(t_{k}\right)x_{1}\left(t_{k}\right)\\ o_{i}^{c}\left(t_{k}\right) & = & \varphi_{i}\left(t_{k}\right)x_{i}\left(t_{k}\right);\;i_{i}^{c}\left(t_{k}\right)=\delta_{i+1}\left(t_{k}\right)x_{i+1}\left(t_{k}\right),\;\;i=1,\ldots ,N-2\\ o_{N-1}^{c}\left(t_{k}\right) & = & \varphi_{N-1}\left(t_{k}\right)x_{N-1}\left(t_{k}\right);\\ i_{N-1}^{c}\left(t_{k}\right) & = & \delta_{N}\left(t_{k}\right)x_{N}\left(t_{k}\right)\\ f_{n}^{e}\left(t_{k}\right) & = & \zeta_{n}\left(t_{k}\right);\;\;f_{n}^{n}\left(t_{k}\right)=\theta_{n}\left(t_{k}\right);\\ g_{n}^{e}\left(t_{k}\right) & = & \kappa_{n}\left(t_{k}\right);\;\;g_{n}^{n}\left(t_{k}\right)=\lambda_{n}\left(t_{k}\right)\\ h_{n}^{e}\left(t_{k}\right) & = & \nu_{n}\left(t_{k}\right);\;\;h_{n}^{n}\left(t_{k}\right)=\pi_{n}\left(t_{k}\right)\\ p_{n}^{e}\left(t_{k}\right) & = & \rho_{n}\left(t_{k}\right);\;\;p_{n}^{n}\left(t_{k}\right)=\tau_{n}\left(t_{k}\right) \end{array}$$

Moreover, a lack of inflow from the (i+1)-th node to the *i*-th node of the channel will be assumed. For the stationary motion of the substance, $x_{i}\left(t_{k+1}\right)=x_{i}\left(t_{k}\right)$, i=0,…,N. The node parameters are assumed to be independent of time: $\sigma_{i}\left(t_{k}\right)=\sigma$; $\mu_{i}\left(t_{k}\right)=\mu_{i}$; $\gamma_{i}\left(t_{k}\right)=\gamma_{i}$; $\epsilon_{i}\left(t_{k}\right)=\epsilon_{i}$; $f_{0}\left(t_{k}\right)=f_{0}$; i=0,…,N. Equation (29) becomes

(31) $$\begin{array}{c} \left[\sigma_{0}-\mu_{0}-\varphi_{0}-\gamma_{0}+\epsilon_{0}\right]x_{0}=0\\ \left[\mu_{i}+\varphi_{i}+\gamma_{i}-\sigma_{i}-\epsilon_{i}\right]x_{i}=\varphi_{i-1}x_{i-1}+\left(-\zeta_{i-1}-\theta_{i-1}+\kappa_{i-1}+\lambda_{i-1}\right),i=1,\ldots ,N-1\\ \left[\mu_{N}+\gamma_{N}-\sigma_{N}-\epsilon_{N}\right]x_{N}=\varphi_{N-1}x_{N-1}+\left(-\zeta_{N-1}-\theta_{N-1}+\kappa_{N-1}+\lambda_{N-1}\right) \end{array}$$

From (31), one obtains the following relationships ($\delta_{i,j}$ denotes the Kronecker delta symbol):

$$\begin{array}{ccc} \varphi_{0} & = & \sigma_{0}-\mu_{0}-\gamma_{0}+\epsilon_{0}, \end{array}$$

$$\begin{array}{ccc} x_{k} & = & x_{0}\prod_{i=1}^{k}\frac{\varphi_{i-1}}{\mu_{i}+\varphi_{i}+\gamma_{i}-\sigma_{i}-\epsilon_{i}}+\\ & & \left(1-\delta_{k1}\right)\sum_{j=1}^{k-1}\left(\frac{\kappa_{j-1}+\lambda_{j-1}-\zeta_{j-1}-\theta_{j-1}}{\mu_{j}+\varphi_{j}+\gamma_{j}-\sigma_{j}-\epsilon_{j}}\prod_{l=j+1}^{k}\frac{\varphi_{l-1}}{\mu_{l}+\varphi_{l}+\gamma_{l}-\sigma_{l}-\epsilon_{l}}\right)+\\ & & \frac{\kappa_{k-1}+\lambda_{k-1}-\zeta_{k-1}-\theta_{k-1}}{\mu_{k}+\varphi_{k}+\gamma_{k}-\sigma_{k}-\epsilon_{k}},\;\;\;k=1,\ldots ,N-1, \end{array}$$

(32) $$\begin{array}{ccc} x_{N} & = & x_{0}\frac{\varphi_{N-1}}{\mu_{N}+\gamma_{N}-\sigma_{N}-\epsilon_{N}}\prod_{i=1}^{N-1}\frac{\varphi_{i-1}}{\mu_{i}+\varphi_{i}+\gamma_{i}-\sigma_{i}-\epsilon_{i}}+\\ & & \frac{\varphi_{N-1}}{\mu_{N}+\gamma_{N}-\sigma_{N}-\epsilon_{N}}\left(1-\delta_{N,2}\right)\sum_{j=1}^{N-2}\left(\frac{\kappa_{j-1}+\lambda_{j-1}-\zeta_{j-1}-\theta_{j-1}}{\mu_{j}+\varphi_{j}+\gamma_{j}-\sigma_{j}-\epsilon_{j}}\prod_{l=j+1}^{N-1}\frac{\varphi_{l-1}}{\mu_{l}+\varphi_{l}+\gamma_{l}-\sigma_{l}-\epsilon_{l}}\right)+\\ & & \frac{\varphi_{N-1}}{\mu_{N}+\gamma_{N}-\sigma_{N}-\epsilon_{N}}\frac{\kappa_{N-2}+\lambda_{N-2}-\zeta_{N-2}-\theta_{N-2}}{\mu_{N-1}+\varphi_{N-1}+\gamma_{N-1}-\sigma_{N-1}-\epsilon_{N-1}}+\frac{\kappa_{N-1}+\lambda_{N-1}-\zeta_{N-1}-\theta_{N-1}}{\mu_{N}+\gamma_{N}-\sigma_{N}-\epsilon_{N}}, \end{array}$$

and

(33) $$\begin{array}{ccc} x & = & \sum_{k=0}^{N}x_{k}=x_{0}\left[1+\sum_{k=1}^{N-1}\prod_{i=1}^{k}\frac{\varphi_{i-1}}{\mu_{i}+\varphi_{i}+\gamma_{i}-\sigma_{i}-\epsilon_{i}}+\frac{\varphi_{N-1}}{\mu_{N}+\gamma_{N}-\sigma_{N}-\epsilon_{N}}\prod_{i=1}^{N-1}\frac{\varphi_{i-1}}{\mu_{i}+\varphi_{i}+\gamma_{i}-\sigma_{i}-\epsilon_{i}}\right]+\\ & & \sum_{k=1}^{N-1}[\left(1-\delta_{k,1}\right)\sum_{j=1}^{k-1}\left(\frac{\kappa_{j-1}+\lambda_{j-1}-\zeta_{j-1}-\theta_{j-1}}{\mu_{j}+\varphi_{j}+\gamma_{j}-\sigma_{j}-\epsilon_{j}}\prod_{l=j+1}^{k}\frac{\varphi_{l-1}}{\mu_{l}+\varphi_{l}+\gamma_{l}-\sigma_{l}-\epsilon_{l}}\right)+\\ & & \frac{\kappa_{k-1}+\lambda_{k-1}-\zeta_{k-1}-\theta_{k-1}}{\mu_{k}+\varphi_{k}+\gamma_{k}-\sigma_{k}-\epsilon_{k}}]+\frac{\varphi_{N-1}}{\mu_{N}+\gamma_{N}-\sigma_{N}-\epsilon_{N}}\left(1-\delta_{N,2}\right)\sum_{j=1}^{N-2}(\frac{\kappa_{j-1}+\lambda_{j-1}-\zeta_{j-1}-\theta_{j-1}}{\mu_{j}+\varphi_{j}+\gamma_{j}-\sigma_{j}-\epsilon_{j}}\times \\ & & \prod_{l=j+1}^{N-1}\frac{\varphi_{l-1}}{\mu_{l}+\varphi_{l}+\gamma_{l}-\sigma_{l}-\epsilon_{l}})+\frac{\varphi_{N-1}}{\mu_{N}+\gamma_{N}-\sigma_{N}-\epsilon_{N}}\frac{\kappa_{N-2}+\lambda_{N-2}-\zeta_{N-2}-\theta_{N-2}}{\mu_{N-1}+\varphi_{N-1}+\gamma_{N-1}-\sigma_{N-1}-\epsilon_{N-1}}+\\ & & \frac{\kappa_{N-1}+\lambda_{N-1}-\zeta_{N-1}-\theta_{N-1}}{\mu_{N}+\gamma_{N}-\sigma_{N}-\epsilon_{N}}. \end{array}$$

Equations (32) and (33) are connected to a class of probability distributions. One can construct a probability distribution $y_{i}=x_{i}/x$ that is connected to the amount of the substance in the channel nodes. $y_{i}$ are probability values of the distribution of a random variable ω: $y_{i}=p\left(\omega =i\right)$, i=0,…,N. One obtains the following for $y_{i}$:

(34) $$\begin{array}{ccc} y_{0} & = & x_{0}/\{x_{0}\left[1+\sum_{k=1}^{N-1}\prod_{i=1}^{k}\frac{\varphi_{i-1}}{\mu_{i}+\varphi_{i}+\gamma_{i}-\sigma_{i}-\epsilon_{i}}+\frac{\varphi_{N-1}}{\mu_{N}+\gamma_{N}-\sigma_{N}-\epsilon_{N}}\prod_{i=1}^{N-1}\frac{\varphi_{i-1}}{\mu_{i}+\varphi_{i}+\gamma_{i}-\sigma_{i}-\epsilon_{i}}\right]+\\ & & \sum_{k=1}^{N-1}[\left(1-\delta_{k,1}\right)\sum_{j=1}^{k-1}\left(\frac{\kappa_{j-1}+\lambda_{j-1}-\zeta_{j-1}-\theta_{j-1}}{\mu_{j}+\varphi_{j}+\gamma_{j}-\sigma_{j}-\epsilon_{j}}\prod_{l=j+1}^{k}\frac{\varphi_{l-1}}{\mu_{l}+\varphi_{l}+\gamma_{l}-\sigma_{l}-\epsilon_{l}}\right)+\\ & & \frac{\kappa_{k-1}+\lambda_{k-1}-\zeta_{k-1}-\theta_{k-1}}{\mu_{k}+\varphi_{k}+\gamma_{k}-\sigma_{k}-\epsilon_{k}}]+\frac{\varphi_{N-1}}{\mu_{N}+\gamma_{N}-\sigma_{N}-\epsilon_{N}}\left(1-\delta_{N,2}\right)\sum_{j=1}^{N-2}(\frac{\kappa_{j-1}+\lambda_{j-1}-\zeta_{j-1}-\theta_{j-1}}{\mu_{j}+\varphi_{j}+\gamma_{j}-\sigma_{j}-\epsilon_{j}}\times \\ & & \prod_{l=j+1}^{N-1}\frac{\varphi_{l-1}}{\mu_{l}+\varphi_{l}+\gamma_{l}-\sigma_{l}-\epsilon_{l}})+\frac{\varphi_{N-1}}{\mu_{N}+\gamma_{N}-\sigma_{N}-\epsilon_{N}}\frac{\kappa_{N-2}+\lambda_{N-2}-\zeta_{N-2}-\theta_{N-2}}{\mu_{N-1}+\varphi_{N-1}+\gamma_{N-1}-\sigma_{N-1}-\epsilon_{N-1}}+\\ & & \frac{\kappa_{N-1}+\lambda_{N-1}-\zeta_{N-1}-\theta_{N-1}}{\mu_{N}+\gamma_{N}-\sigma_{N}-\epsilon_{N}}\}, \end{array}$$

(35) $$\begin{array}{ccc} y_{k} & = & \{x_{0}\prod_{i=1}^{k}\frac{\varphi_{i-1}}{\mu_{i}+\varphi_{i}+\gamma_{i}-\sigma_{i}-\epsilon_{i}}+\left(1-\delta_{k1}\right)\sum_{j=1}^{k-1}\left(\frac{\kappa_{j-1}+\lambda_{j-1}-\zeta_{j-1}-\theta_{j-1}}{\mu_{j}+\varphi_{j}+\gamma_{j}-\sigma_{j}-\epsilon_{j}}\prod_{l=j+1}^{k}\frac{\varphi_{l-1}}{\mu_{l}+\varphi_{l}+\gamma_{l}-\sigma_{l}-\epsilon_{l}}\right)+\\ & & \frac{\kappa_{k-1}+\lambda_{k-1}-\zeta_{k-1}-\theta_{k-1}}{\mu_{k}+\varphi_{k}+\gamma_{k}-\sigma_{k}-\epsilon_{k}}\}/\{x_{0}[1+\sum_{k=1}^{N-1}\prod_{i=1}^{k}\frac{\varphi_{i-1}}{\mu_{i}+\varphi_{i}+\gamma_{i}-\sigma_{i}-\epsilon_{i}}+\\ & & \frac{\varphi_{N-1}}{\mu_{N}+\gamma_{N}-\sigma_{N}-\epsilon_{N}}\prod_{i=1}^{N-1}\frac{\varphi_{i-1}}{\mu_{i}+\varphi_{i}+\gamma_{i}-\sigma_{i}-\epsilon_{i}}]+\\ & & \sum_{k=1}^{N-1}\left[\left(1-\delta_{k,1}\right)\sum_{j=1}^{k-1}\left(\frac{\kappa_{j-1}+\lambda_{j-1}-\zeta_{j-1}-\theta_{j-1}}{\mu_{j}+\varphi_{j}+\gamma_{j}-\sigma_{j}-\epsilon_{j}}\prod_{l=j+1}^{k}\frac{\varphi_{l-1}}{\mu_{l}+\varphi_{l}+\gamma_{l}-\sigma_{l}-\epsilon_{l}}\right)+\frac{\kappa_{k-1}+\lambda_{k-1}-\zeta_{k-1}-\theta_{k-1}}{\mu_{k}+\varphi_{k}+\gamma_{k}-\sigma_{k}-\epsilon_{k}}\right]+\\ & & \frac{\varphi_{N-1}}{\mu_{N}+\gamma_{N}-\sigma_{N}-\epsilon_{N}}\left(1-\delta_{N,2}\right)\sum_{j=1}^{N-2}\left(\frac{\kappa_{j-1}+\lambda_{j-1}-\zeta_{j-1}-\theta_{j-1}}{\mu_{j}+\varphi_{j}+\gamma_{j}-\sigma_{j}-\epsilon_{j}}\prod_{l=j+1}^{N-1}\frac{\varphi_{l-1}}{\mu_{l}+\varphi_{l}+\gamma_{l}-\sigma_{l}-\epsilon_{l}}\right)+\\ & & \frac{\varphi_{N-1}}{\mu_{N}+\gamma_{N}-\sigma_{N}-\epsilon_{N}}\frac{\kappa_{N-2}+\lambda_{N-2}-\zeta_{N-2}-\theta_{N-2}}{\mu_{N-1}+\varphi_{N-1}+\gamma_{N-1}-\sigma_{N-1}-\epsilon_{N-1}}+\frac{\kappa_{N-1}+\lambda_{N-1}-\zeta_{N-1}-\theta_{N-1}}{\mu_{N}+\gamma_{N}-\sigma_{N}-\epsilon_{N}}\},\\ & & k=1,\ldots ,N-1, \end{array}$$

(36) $$\begin{array}{ccc} y_{N} & = & \{x_{0}\frac{\varphi_{N-1}}{\mu_{N}+\gamma_{N}-\sigma_{N}-\epsilon_{N}}\prod_{i=1}^{N-1}\frac{\varphi_{i-1}}{\mu_{i}+\varphi_{i}+\gamma_{i}-\sigma_{i}-\epsilon_{i}}+\\ & & \frac{\varphi_{N-1}}{\mu_{N}+\gamma_{N}-\sigma_{N}-\epsilon_{N}}\left(1-\delta_{N,2}\right)\sum_{j=1}^{N-2}\left(\frac{\kappa_{j-1}+\lambda_{j-1}-\zeta_{j-1}-\theta_{j-1}}{\mu_{j}+\varphi_{j}+\gamma_{j}-\sigma_{j}-\epsilon_{j}}\times \prod_{l=j+1}^{N-1}\frac{\varphi_{l-1}}{\mu_{l}+\varphi_{l}+\gamma_{l}-\sigma_{l}-\epsilon_{l}}\right)+\\ & & \frac{\varphi_{N-1}}{\mu_{N}+\gamma_{N}-\sigma_{N}-\epsilon_{N}}\frac{\kappa_{N-2}+\lambda_{N-2}-\zeta_{N-2}-\theta_{N-2}}{\mu_{N-1}+\varphi_{N-1}+\gamma_{N-1}-\sigma_{N-1}-\epsilon_{N-1}}+\frac{\kappa_{N-1}+\lambda_{N-1}-\zeta_{N-1}-\theta_{N-1}}{\mu_{N}+\gamma_{N}-\sigma_{N}-\epsilon_{N}}\}/\\ & & \{x_{0}\left[1+\sum_{k=1}^{N-1}\prod_{i=1}^{k}\frac{\varphi_{i-1}}{\mu_{i}+\varphi_{i}+\gamma_{i}-\sigma_{i}-\epsilon_{i}}+\frac{\varphi_{N-1}}{\mu_{N}+\gamma_{N}-\sigma_{N}-\epsilon_{N}}\prod_{i=1}^{N-1}\frac{\varphi_{i-1}}{\mu_{i}+\varphi_{i}+\gamma_{i}-\sigma_{i}-\epsilon_{i}}\right]+\\ & & \sum_{k=1}^{N-1}[\left(1-\delta_{k,1}\right)\sum_{j=1}^{k-1}\left(\frac{\kappa_{j-1}+\lambda_{j-1}-\zeta_{j-1}-\theta_{j-1}}{\mu_{j}+\varphi_{j}+\gamma_{j}-\sigma_{j}-\epsilon_{j}}\prod_{l=j+1}^{k}\frac{\varphi_{l-1}}{\mu_{l}+\varphi_{l}+\gamma_{l}-\sigma_{l}-\epsilon_{l}}\right)+\\ & & \frac{\kappa_{k-1}+\lambda_{k-1}-\zeta_{k-1}-\theta_{k-1}}{\mu_{k}+\varphi_{k}+\gamma_{k}-\sigma_{k}-\epsilon_{k}}]+\frac{\varphi_{N-1}}{\mu_{N}+\gamma_{N}-\sigma_{N}-\epsilon_{N}}\left(1-\delta_{N,2}\right)\sum_{j=1}^{N-2}(\frac{\kappa_{j-1}+\lambda_{j-1}-\zeta_{j-1}-\theta_{j-1}}{\mu_{j}+\varphi_{j}+\gamma_{j}-\sigma_{j}-\epsilon_{j}}\times \\ & & \prod_{l=j+1}^{N-1}\frac{\varphi_{l-1}}{\mu_{l}+\varphi_{l}+\gamma_{l}-\sigma_{l}-\epsilon_{l}})+\frac{\varphi_{N-1}}{\mu_{N}+\gamma_{N}-\sigma_{N}-\epsilon_{N}}\frac{\kappa_{N-2}+\lambda_{N-2}-\zeta_{N-2}-\theta_{N-2}}{\mu_{N-1}+\varphi_{N-1}+\gamma_{N-1}-\sigma_{N-1}-\epsilon_{N-1}}+\\ & & \frac{\kappa_{N-1}+\lambda_{N-1}-\zeta_{N-1}-\theta_{N-1}}{\mu_{N}+\gamma_{N}-\sigma_{N}-\epsilon_{N}}\}. \end{array}$$

The following interesting theorem can be proved [293].

**Theorem** **1.** *Any truncated discrete probability distribution of the random variable ω that can take values 0,1,…,N is a specific case of the probability distribution (34)–(36).*

The corresponding probability distribution for a channel possessing an infinite number of nodes is [293]:

(37) $$\begin{array}{ccc} y_{0} & = & x_{0}/\{x_{0}\left[1+\sum_{k=1}^{\infty }\prod_{i=1}^{k}\frac{\varphi_{i-1}}{\mu_{i}+\varphi_{i}+\gamma_{i}-\sigma_{i}-\epsilon_{i}}\right]+\\ & & \sum_{k=1}^{\infty }\left[\left(1-\delta_{k,1}\right)\sum_{j=1}^{k-1}\left(\frac{\kappa_{j-1}+\lambda_{j-1}-\zeta_{j-1}-\theta_{j-1}}{\mu_{j}+\varphi_{j}+\gamma_{j}-\sigma_{j}-\epsilon_{j}}\prod_{l=j+1}^{k}\frac{\varphi_{l-1}}{\mu_{l}+\varphi_{l}+\gamma_{l}-\sigma_{l}-\epsilon_{l}}\right)+\frac{\kappa_{k-1}+\lambda_{k-1}-\zeta_{k-1}-\theta_{k-1}}{\mu_{k}+\varphi_{k}+\gamma_{k}-\sigma_{k}-\epsilon_{k}}\right]\},\\ y_{k} & = & \{x_{0}\prod_{i=1}^{k}\frac{\varphi_{i-1}}{\mu_{i}+\varphi_{i}+\gamma_{i}-\sigma_{i}-\epsilon_{i}}+\left(1-\delta_{k1}\right)\sum_{j=1}^{k-1}\left(\frac{\kappa_{j-1}+\lambda_{j-1}-\zeta_{j-1}-\theta_{j-1}}{\mu_{j}+\varphi_{j}+\gamma_{j}-\sigma_{j}-\epsilon_{j}}\prod_{l=j+1}^{k}\frac{\varphi_{l-1}}{\mu_{l}+\varphi_{l}+\gamma_{l}-\sigma_{l}-\epsilon_{l}}\right)+\\ & & \frac{\kappa_{k-1}+\lambda_{k-1}-\zeta_{k-1}-\theta_{k-1}}{\mu_{k}+\varphi_{k}+\gamma_{k}-\sigma_{k}-\epsilon_{k}}\}/\{x_{0}\left[1+\sum_{k=1}^{\infty }\prod_{i=1}^{k}\frac{\varphi_{i-1}}{\mu_{i}+\varphi_{i}+\gamma_{i}-\sigma_{i}-\epsilon_{i}}\right]+\\ & & \sum_{k=1}^{\infty }[\left(1-\delta_{k,1}\right)\sum_{j=1}^{k-1}\left(\frac{\kappa_{j-1}+\lambda_{j-1}-\zeta_{j-1}-\theta_{j-1}}{\mu_{j}+\varphi_{j}+\gamma_{j}-\sigma_{j}-\epsilon_{j}}\prod_{l=j+1}^{k}\frac{\varphi_{l-1}}{\mu_{l}+\varphi_{l}+\gamma_{l}-\sigma_{l}-\epsilon_{l}}\right)+\\ & & \frac{\kappa_{k-1}+\lambda_{k-1}-\zeta_{k-1}-\theta_{k-1}}{\mu_{k}+\varphi_{k}+\gamma_{k}-\sigma_{k}-\epsilon_{k}}]\},\;\;k=1,\ldots ,\infty \end{array}$$

The following theorem can be proved [293].

**Theorem** **2.** *Any discrete probability distribution of the random variable ω that can take the values 0,1,… is a specific case of the probability distribution (37).*

The discrete model of substance flow in a network of channels considered here leads to an analytical relationship for the distribution of the substance for a stationary flow in a channel containing an arbitrary nonnegative number of nodes (this number can be finite or infinite). This distribution possesses all possible distributions of a random variable that can take the discrete values 0,1,…,N as specific cases. Here, numerous famous named distributions are included. In addition, entire families of distributions, such as the families of distributions of Katz, Ord, Kemp, etc. [294,295,296,297,298,299,300,301,302,303,304,305,306,307], are included.

The theory discussed above has many applications. The problems of human migration stimulated the beginning of this research [195,196,197,198,199]. In [195], a mathematical model of a migration channel was discussed on the basis of the truncated Waring distribution. The model was applied for the case of motion of migrants through a channel consisting of a finite number of countries or cities. The number of migrants in this channel depended heavily on the number of migrants that entered the channel through the country of entrance. If the final destination country is very popular, then according to the model, a large percentage of migrants might concentrate there. A theory for the motion of two substances in a channel was discussed in [196]. In [197], the problem of the motion of a substance in a channel of a network was studied for the case of a channel that had two arms. For the case of the stationary flow of the substance, analytical relationships for the substance distribution in the nodes of the arms of the channel were obtained. These distributions were in connection with technological applications of the model, such as the motion of substances such as water in complex technological facilities. In [198,199], the motion of a substance was studied in a channel of a network that had three arms. For the case of the stationary flow of a substance, one obtains probability distributions for the amount of the substance in the nodes of the channel that contain famous long-tail distributions, such as the Yule–Simon distribution and the Zipf distribution, as specific cases. The obtained results were discussed from the point of view of the motion of the substance in a complex technological system and for the case of human migration.

In addition, the above theory can be used to study numerous additional situations in the area of substance flow in systems that possess network structures.

## 4. Connection between the Theory of Flows in Networks and the Theory of Growth of Random Networks

One of the specific cases of the above general discrete model has an interesting relationship with the theory of growth of random networks [308]. For this specific case, one considers the integer *l*, which can take values 0,1,…, and then one labels the nodes of the channel as l,l+1,…. The substance moves along nodes of the channel. The following four events can happen in a unit of time. The substance in a node may remain in the same node. The second possible event is the motion of the substance from node *m* to node m+1. The third possible event is leakage, where the substance may leave the channel. The final possible event is the entrance of a substance from the environment into the node under consideration.

The following notations are used. $i_{n}^{e}\left(t_{u}\right)$/$o_{n}^{e}\left(t_{u}\right)$ are the amounts of inflow/outflow of the substance from the environment to the *n*-th mode/from the *n*-th node of the channel to the environment. $o_{n}^{cn}\left(t_{u}\right)$ is the outflow of the substance from the *n*-th to the (n+1)-th node. $i_{n}^{cn}\left(t_{u}\right)$ is the inflow of the substance from the (n+1) node to the *n*-th node.

Node *l* is the point of entrance of the substance. The substance moves from node *l* in the direction of nodes with numbers l+1, …. Leakage of the substance is possible in any of the nodes of the channel. The corresponding model equations are:

(38) $$\begin{array}{ccc} x_{l}\left(t_{u+1}\right) & = & x_{l}\left(t_{u}\right)+i_{l}^{e}\left(t_{u}\right)-o_{l}^{cn}\left(t_{u}\right)-o_{l}^{e}\left(t_{u}\right)\\ x_{i}\left(t_{u+1}\right) & = & x_{i}\left(t_{u}\right)+o_{i-1}^{cn}\left(t_{u}\right)-o_{i}^{cn}\left(t_{u}\right)-o_{i}^{e}\left(t_{u}\right),\;\;i=l+1,\ldots \end{array}$$

The following specific case for relationships of the quantities is studied below: $i_{l}^{e}\left(t_{u}\right)=\sigma \left(t_{u}\right)x_{l}\left(t_{u}\right);o_{l}^{cn}\left(t_{u}\right)=f_{l}\left(t_{u}\right)x_{l}\left(t_{u}\right)$; $o_{l}^{e}\left(t_{u}\right)=\epsilon_{l}\left(t_{u}\right)x_{l}\left(t_{u}\right);o_{i-1}^{cn}\left(t_{u}\right)=f_{i-1}\left(t_{u}\right)x_{i-1}\left(t_{u}\right)$; $o_{i}^{cn}\left(t_{u}\right)=f_{i}\left(t_{u}\right)x_{i}\left(t_{u}\right);o_{i}^{e}\left(t_{u}\right)=\epsilon_{i}\left(t_{u}\right)x_{i}\left(t_{u}\right)$; i=l+1,…. We assume that: $\sigma (t_{u})=\sigma$; $\alpha_{i}\left(t_{u}\right)=\alpha_{i}$, i=l,…; $\epsilon_{i}\left(t_{u}\right)=\epsilon_{i}$, i=l,…, $f_{i}\left(t_{u}\right)=f_{i}$, i=l,…. Then, (38) becomes:

(39) $$\begin{array}{ccc} x_{l}\left(t_{u+1}\right) & = & x_{l}\left(t_{u}\right)+\sigma x_{l}\left(t_{u}\right)-f_{l}x_{l}\left(t_{u}\right)-\epsilon_{l}x_{l}\left(t_{u}\right)\\ x_{i}\left(t_{u+1}\right) & = & x_{i}\left(t_{u}\right)+f_{i-1}x_{i-1}\left(t_{u}\right)-f_{i}x_{i}\left(t_{u}\right)-\epsilon_{i}x_{i}\left(t_{u}\right)\;\;i=l+1,\ldots \end{array}$$

For the stationary state, $x_{i}\left(t_{u}\right)=x_{i}^{*}$ (occurs when $x_{i}\left(t_{u+1}\right)=x_{i}\left(t_{u}\right)$), and one obtains the following from the system of Equation (39) ($\sigma =f_{l}+\epsilon_{l}$, and because of this, $x_{l}^{*}$ becomes a free parameter): $x_{i}^{*}=\frac{f_{i-1}}{f_{i}+\epsilon_{i}}x_{i-1}^{*},\;\;\;i=l+1,\ldots$ Then,

(40) $$\begin{array}{c} x_{i}^{*}=x_{l}^{*}\prod_{j=l+1}^{i}\frac{f_{j-1}}{f_{j}+\epsilon_{j}},\;\;\;i=l+1,\ldots \end{array}$$

The amount of the substance in all of the channel nodes is $x^{*}=x_{l}^{*}\left[1+\sum_{k=l+1}^{\infty }\prod_{j=l+1}^{k}\frac{f_{j-1}}{f_{j}+\epsilon_{j}}\right].$ $p_{i}=x_{i}^{*}/x^{*}$ is the probability distribution of the amount of the substance along the channel nodes. One obtains

(41) $$\begin{array}{c} p_{l}=\frac{1}{1+\sum_{k=l+1}^{\infty }\prod_{j=l+1}^{k}\frac{f_{j-1}}{f_{j}+\epsilon_{j}}},\;\;\;p_{i}=\frac{\prod_{j=l+1}^{i}\frac{f_{j-1}}{f_{j}+\epsilon_{j}}}{1+\sum_{k=l+1}^{\infty }\prod_{j=l+1}^{k}\frac{f_{j-1}}{f_{j}+\epsilon_{j}}},\;\;\;i=l+1,\ldots \end{array}$$

The specific case of (41) in which $\epsilon_{i}=\epsilon$ is of interest for us in the following. In this case, (41) is

(42) $$\begin{array}{c} p_{l}=\frac{1}{1+\sum_{k=l+1}^{\infty }\prod_{j=l+1}^{k}\frac{f_{j-1}}{f_{j}+\epsilon }},\;\;\;p_{i}=\frac{\prod_{j=l+1}^{i}\frac{f_{j-1}}{f_{j}+\epsilon }}{1+\sum_{k=l+1}^{\infty }\prod_{j=l+1}^{k}\frac{f_{j-1}}{f_{j}+\epsilon }},\;\;\;i=l+1,\ldots \end{array}$$

Specific values of $f_{i}$ lead to various distributions. For the case in which $f_{i}=\alpha =\mathrm{const}$ (α>0) (case of stationary flow where the transfer of the substance between the channel nodes is the same for all nodes of the channel), (41) is reduced to

(43) $$p_{l}=\frac{1}{1+\alpha /\epsilon };\;\;\;p_{i}=\frac{{\left(\frac{\alpha }{\alpha +\epsilon }\right)}^{i-l}}{1+\alpha /\epsilon },i=l+1,\ldots$$

Equation (43) is a geometric distribution: $p_{i}=q{\left(1-q\right)}^{i-l},\;\;\;q=\frac{\epsilon }{\alpha +\epsilon },\;\;\;i=l,\ldots$.

The next possibility is $f_{j+r}=f_{j}+\beta r$, where β>0 and *r* is an integer. A solution of the above relationship is $f_{j}=\alpha +\left(j-s\right)\beta >0.$ *s* is an integer. An additional assumption is that s<l+1. For the specific relationships a=α/β and b=ϵ/β, we obtain the probability distribution

(44) $$\begin{array}{ccc} p_{i} & = & p_{l}\prod_{j=l+1}^{i}\frac{f_{j-1}}{f_{j}+\epsilon }=p_{l}\frac{f_{l}\ldots f_{i-1}}{\left(f_{l+1}+\epsilon \right)\ldots \left(f_{i}+\epsilon \right)}=p_{l}\frac{\left(a+l-s)\ldots (a+i-s-1\right)}{\left(a+b+l+1-s)\ldots (a+b+i-s\right)}=\\ & & p_{l}\frac{\Gamma (a+i-s)}{\Gamma (a+l-s)}\frac{\Gamma (a+b+l+1-s)}{\Gamma (a+b+i+1-s)}. \end{array}$$

In (44), Γ(x) is the Gamma function. Equation (44) can be written by means of the Beta function: $p_{i}=p_{l}\frac{B(a+i-s,b+1)}{B(a+l-s,b+1)}.$ The asymptotic behavior of the Beta function for large values of *x* is $B\left(x,y\right)≃x^{-y}\Gamma \left(y\right).$ Thus, $p_{i}≃p_{l}\frac{\Gamma (b+1)}{B(a+l-s,b+1)}{\left(a+i-s\right)}^{-(b+1)}$. For large enough values of *i*,

(45) $$p_{i}\propto i^{-(b+1)}.$$

Equation (45) exhibits a power law behavior of $p_{i}$ for large values of *i*.

The value of the parameter b=ϵ/β controls the balance between the leakage (accounted for by ϵ) and the transfer of the substance between the channel nodes (accounted for by β). For example, if ϵ=2β, then the asymptotic power law becomes $p_{i}≃1/i^{3}$.

We can consider many other cases for $f_{i}$ [308]. Selected results are given in the following. First of all, let $f_{j+r}=f_{j}+\epsilon$ (*r* is an integer). In addition, β>0 and α>−(l−s)β. The solution is $f_{j}=\alpha +\left(j-s\right)\beta ,\;\;\;\beta =\epsilon /r.$ Here, α and β are real parameters and *s* is an integer. The assumption r<l+1 and s<l+1 and the relationships a=α/β; b=ϵ/β lead to an asymptotic power law relationship: $p_{i}\propto i^{-(1+r)}.$ *r* must be an integer. In addition, r=ϵ/β. The lack of leakage (ϵ=0) is equivalent to $p_{i}∼1/i$. The last power law relationship estimates the maximum possible amount of the substance in the *i*-th channel node.

Next, we consider the case in which $f_{j}=j^{\gamma }$, ϵ>0. Here, ϵ and γ are constants. It follows from (41) that:

(46) $$p_{i}=p_{l}\frac{l^{\gamma }\ldots {\left(i-1\right)}^{\gamma }}{\left[{\left(l+1\right)}^{\gamma }+\epsilon \right]\ldots \left[i^{\gamma }+\epsilon \right]}=\frac{l^{\gamma }}{i^{\gamma }}\frac{1}{\left[1+\epsilon /{\left(l+1\right)}^{\gamma }\right]\ldots \left[1+\epsilon /i^{\gamma }\right]}$$

Equation (46) can be written as $p_{i}=\frac{p_{l}l^{\gamma }}{i^{\gamma }}\exp\left\{-\sum_{j=l+1}^{i}\ln\left(1+\frac{\epsilon }{j^{\gamma }}\right)\right\}$ An approximate evaluation of the last relationship [308] leads to

(47) $$p_{i}∼i^{-\gamma }\exp\left\{\sum_{s=1}^{m}\frac{{\left(-1\right)}^{s}}{s}\epsilon^{s}\frac{i^{1-\gamma s}}{1-\gamma s}\right\},\;\;\;\frac{1}{m+1}<\gamma <\frac{1}{m},m=1,\ldots$$

Finally, we consider the case in which $f_{j}=j^{\gamma }$, γ<0, ϵ>0. The specific case $f_{j}=1/j^{-\gamma }$ is related to the motion of the substance between the nodes, which decreases with increasing node number *j*. In this case, we set $\hat{\gamma }=-\gamma$. The result for $p_{i}$ is

(48) $$p_{i}∼p_{l}\frac{\Gamma (l)}{\Gamma {\left(i\right)}^{\hat{\gamma }}\epsilon^{i-l}}\exp\left\{\sum_{s=1}^{m}\frac{{\left(-1\right)}^{s}}{s}\frac{1}{\epsilon^{s}}\frac{i^{1-\hat{\gamma }s}}{1-\hat{\gamma }s}\right\},\;\;\;\frac{1}{m+1}<\hat{\gamma }<\frac{1}{m},m=1,\ldots$$

The connection between the theory of the motion of a substance in a channel and the theory of growth of random networks is that the probability connected to the distribution of a substance for the stationary motion of that substance in a channel contains, as a specific case, the degree distribution for the nodes of large degrees in a growing random network. In several more words, let us set $\epsilon_{i}=\epsilon$, l=c, ϵ=μ/c, $f_{i}=a_{i}$, and

(49) $$a_{c}=\epsilon \sum_{k=c+1}^{\infty }\prod_{j=c+1}^{k}\frac{a_{j-1}}{a_{j}+\epsilon },$$

Then, (42) becomes

(50) $$p_{c}=\frac{\mu /c}{a_{c}+\mu /c},\;\;\;p_{k}=\frac{a_{k-1}}{a_{k}+\mu /c}p_{k-1},\;\;k=c+1,\cdots .$$

Equation (49) defines $a_{c}$ for the case of the motion of a substance in a channel. For the case of fixed $a_{c}$, Equation (49) is an equation for ϵ. Equation (49) can be rewritten as

(51) $$\sum_{k=c}^{\infty }\frac{\mu }{ca_{k}}\prod_{j=c}^{k}{\left(1+\frac{\mu }{ca_{j}}\right)}^{-1}=\sum_{k=c}^{\infty }p_{k}=1.$$

Equation (51) is an equation for μ. The specific case

(52) $$\mu =\sum_{i=c}^{\infty }p_{i}a_{i},$$

is of interest for this discussion. Taking into account that $p_{i}=\frac{\mu }{ca_{i}}\prod_{j=c}^{i}{\left(1+\frac{\mu }{ca_{j}}\right)}^{-1}$, (52) leads to a simplification of (51):

(53) $$\sum_{k=c}^{\infty }\prod_{j=c}^{k}{\left(1+\frac{\mu }{ca_{j}}\right)}^{-1}=c$$

The relationships (49)–(53) are the connection between the theory of growth of random networks and the theory of the flow of a substance in a network channel [309,310,311]. $a_{i}$ is the attachment kernel, which gives the (nonlinear in the general case) dependence of the probability of obtaining a new node of degree *i*. The attachment kernel has the same value for all nodes of the same degree. A basic assumption of the theory is that the average number of edges of the nodes attached to the growing network is *c*. The theory describes a case with a very large size of the growing network. The average value of the attachment kernels with respect to the number of edges is $\mu =\sum_{i}a_{i}p_{i}$. This is the parameter μ from (49)–(53).

For the case of the stationary motion of a substance in a channel, ϵ=μ/c. ϵ is the leakage parameter, and it is equal to the ratio between the average number of edges of the nodes attached to the growing network and the averaged attachment kernel. Another part of the connection is given by the relationship $f_{i}=a_{i}$; in other words, the parameter $f_{i}$, which regulates the flow of the substance into the nodes, is analogous to the attachment kernel in the model of a growing network.

Results for specific cases of growing random networks with the connection to the above results from the theory of substance flow in a network are given in the following. For the specific case in which $f_{i}=1$, there is an attachment without a preference. The solution of (53) is $\mu =\frac{\alpha c}{c-1}$. Then, $\epsilon =\mu /c=\frac{\alpha }{c-1}$. The distribution (43) becomes (l=c): $p_{c}=\frac{1}{c},\;\;\;p_{i}=\frac{{\left(c-1\right)}^{i-c}}{c^{i-c+1}},i=c+1,\ldots .$ There is no dependence on α in this distribution. For i>>c, $p_{i}∼\frac{1}{c}{\left(1-\frac{1}{c}\right)}^{i}.$

Several results for the growth of a random network in the presence of preferential attachment are given in the following. The first case is $a_{i}∼i$. For this case, $f_{i}=a_{i}=\alpha_{i}+\left(i-c\right)\beta_{i}$, i=c+1,…. $\beta_{i}$ and $\alpha_{i}$ are constants. Let us choose $\alpha_{i}=\alpha$, $\beta_{i}=\beta$, and α/β=c. The probability of attachment to a node of degree *i* is $Pr_{i}=\frac{\alpha +(i-c)\beta }{\sum_{j}\left[\alpha +j\beta \right]}=\frac{i}{\sum_{j}j}$. From (53), one obtains for the case in which μ=σcβ and σ is an integer:

(54) $$c=\sum_{k=c}^{\infty }\prod_{j=c}^{k}{\left[1+\frac{\sigma }{c+j}\right]}^{-1}=\sum_{k=c}^{\infty }\frac{c\ldots (c+\sigma -1)}{\left(k+1)\ldots (k+\sigma \right)},\;\;\;\sigma =2,\ldots$$

σ=2 is the solution of (54). Then, μ=2βc, and the distribution (41) becomes

(55) $$\begin{array}{ccc} p_{c} & = & \frac{2}{c+2};\\ p_{i} & = & p_{c}\prod_{k=c+1}^{i}\frac{\alpha +(k-c-1)\beta }{\alpha +(k-c)\beta +\mu /c}=\frac{2}{c+2}\prod_{k=c+1}^{i}\frac{k-1}{k+2}=\frac{2c(c+1)}{i(i+1)(i+2)},\;\;\;i=c+1,\ldots \end{array}$$

For asymptotic values of *i*, $p_{i}$ approaches the power law $p_{i}\approx i^{-3}$.

Another case is that in which $\alpha_{i}=\alpha$, $\beta_{i}=\beta$, and α/β≠c. Then, $a_{i}=\alpha_{i}+\left(i-c\right)\beta_{i}=\alpha +\left(i-c\right)\beta$. We set a=α/β and b=μ/(βc). Equation (41) becomes ($a_{c}=\alpha$):

(56) $$p_{c}=\frac{b}{a+b},\;\;\;p_{i}=p_{c}\frac{{\left(a\right)}^{\left[i-c-1\right]}}{{\left(a+b\right)}^{\left[i-c\right]}},i=c+1,\ldots$$

In (56), ${\left(u\right)}^{\left[j\right]}=\frac{(u+j)!}{u!}=\left(u+j\right)\ldots \left(u+1\right)$ (*u*—real positive number, *j*—integer). Equation (56) is the Waring distribution for c=0. Thus, for the problem of a growing random network, we arrive at a specific case of the Waring distribution. The case is specific, as the condition (53) has to be satisfied.

The distribution (56) can be written with Gamma functions: $p_{c}=\frac{b}{a+b}$, $p_{i}=\frac{b}{a+b}\frac{\Gamma (a+i-c)}{\Gamma (a)}\frac{\Gamma (a+b)}{\Gamma (i-c+a+b+1)},i=c+1,\ldots$ Here, μ=bβc, and this satisfies (53). The behavior of the distribution’s asymptotic values of *i* is $p_{i}≃\frac{a\Gamma (a+b+1)}{{\left(a+b\right)}^{2}\Gamma \left(a\right)}{\left(i-c\right)}^{-(b+1)}.$ The asymptotic behavior of $p_{i}$ for very large values of *i* is

(57) $$p_{i}\propto i^{-(b+1)}$$

For the Barabasi–Albert case, b=2. Thus, $p_{i}\propto i^{-3}$.

The next specific case is that in which $f_{j}=j^{\gamma }$ and ϵ>0. We set $f_{i}=a_{i}=i^{\gamma }$. 0<γ<1 is a constant [309]. From (42), one obtains (ϵ=μ/c; l=c): $p_{i}=\frac{p_{c}c^{\gamma }}{i^{\gamma }}\exp\left\{-\sum_{j=c+1}^{i}\ln\left(1+\frac{\mu }{cj^{\gamma }}\right)\right\}$. By the assumption that $\mu /(cj^{\gamma })<<1$, $\log\left(1+\frac{\epsilon }{j^{\gamma }}\right)$ can be represented as a Taylor series. Thus, $p_{i}=\frac{p_{c}c^{\gamma }}{i^{\gamma }}\exp\left\{\sum_{s=1}^{\infty }\frac{{\left(-1\right)}^{s}}{s}{\left(\frac{\mu }{c}\right)}^{s}\sum_{j=l+1}^{i}j^{-\gamma s}\right\}.$ An approximate evaluation of this relationship leads to

(58) $$p_{i}∼i^{-\gamma }\exp\left\{\sum_{s=1}^{m}\frac{{\left(-1\right)}^{s}}{s}{\left(\frac{\mu }{c}\right)}^{s}\frac{i^{1-\gamma s}}{1-\gamma s}\right\},\;\;\;\frac{1}{m+1}<\gamma <\frac{1}{m},m=1,\ldots$$

Let us consider as an example 1/2<γ<1. Then, we obtain for $p_{i}$:

(59) $$p_{i}∼i^{-\gamma }\exp\left(-\frac{\mu i^{1-\gamma }}{c(1-\gamma )}\right),$$

For large values of *i*, (59) describes a stretched exponential distribution.

Finally, let $f_{j}=j^{\gamma }$, γ<0, and ϵ>0; in addition, $\hat{\gamma }=-\gamma$. The result for $p_{i}$ is

(60) $$p_{i}∼p_{c}\frac{\Gamma (c)}{\Gamma {\left(i\right)}^{\hat{\gamma }}{\left(\mu /c\right)}^{i-c}}\exp\left\{\sum_{s=1}^{m}\frac{{\left(-1\right)}^{s}}{s}\frac{1}{{\left(\mu /c\right)}^{s}}\frac{i^{1-\hat{\gamma }s}}{1-\hat{\gamma }s}\right\},\;\;\;\frac{1}{m+1}<\hat{\gamma }<\frac{1}{m},m=1,\ldots ,$$

The difference with respect to the case γ>0 is connected to the first term from the right-hand side of (60). The corresponding term in (58) is proportional to $i^{-\gamma }$. The term in (60) is proportional to $\Gamma {\left(i\right)}^{-\hat{\gamma }}{\left(\mu /c\right)}^{-(i-c)}$, which, for the same values of the parameters γ and $\hat{\gamma }$, is larger than $i^{-\gamma }$. Thus, the expected number of nodes of a large degree for γ<0 is smaller than the expected number of nodes of the same degree from γ>0.

## 5. Concluding Remarks

In this review paper, we considered selected aspects from the research on flows in networks. A list of references containing basic results and interesting applications was given. The research area of network flows is very large, and this list of references covers only a small part of the research since the publication of the classic book by Ford and Fulkerson. The focus of this article is connected to models of network flows based on differential and difference equations. There are many such models, and we chose to mention only a few of them. These were basic macroscopic models of vehicular traffic, data flows in communication networks, flows in open channels, supply chain networks, and gas flow in pipeline networks.

A large part of this paper considered flows in channels of a network. The channels could be simple ones, such as a channel with a single arm. There are much more complicated channels that possess many arms. A mathematical model of substance flow in such a channel that was based on differential equations was presented in the text. The focus was on obtaining analytical results, and this was the reason for considering stationary substance flow in the channel. Many long-tailed probability distributions connected to the distribution of a substance in the channel nodes can be derived with this model.

The flow of a substance in a channel of a network can also be modeled by using difference equations. A basic model was discussed in this paper, and the emphasis was on the stationary flow of the substance. For this case, analytical results could be obtained. It was shown that the general form of the distribution of the substance in the channel that was obtained contained many named probability distributions, such as the probability distributions of Zifp, Yule–Simon, Waring, the binomial distribution, and entire classes of probability distributions (for example, the classes of probability distributions of Katz, Ord, Kemp, etc.), as specific cases. It was the consequence of a theorem that the derived probability distribution contained any probability distribution of a random variable that could take the values of 0,1,… as a specific case.

The importance of the obtained results was shown by means of the relation between the theories of (i) stationary flow of a substance in a network channel and (ii) the growth of random networks. The model equations for the stationary motion of a substance in the channel have, as a specific case, model equations for changes in the degrees of the nodes of a large and growing random network. In such a way, one can relate the probability distributions for different cases of stationary motion of a substance in a channel to the probability distributions for the node distributions in growing random networks (including the preferential attachment case studied by Barabasi and Albert).

All of the above shows that research on flows in networks is very promising from the point of view of mathematics and from the point of view of its applications to natural and social systems. We hope that this feature paper will contribute to an increase in the interest in this research and to its practical applications.

## References

[1] Ford L.D., Fulkerson D.R. (1962). Flows in Networks.

[2] Boykov Y., Veksler O., Zabih R. (2001). Fast Approximate Energy Minimization via Graph Cuts. IEEE Trans. Pattern Anal. Mach. Intell..

[3] Boykov Y., Funka-Lea G. (2006). Graph Cuts and Efficient N-D Image Segmentation. Int. J. Comput. Vis..

[4] Cheung G., Magli E., Tanaka Y., Ng M.K. (2018). Graph Spectral Image Processing. Proc. IEEE.

[5] Kolmogorov V., Zabih R. Computing Visual Correspondence with Occlusions Using Graph Cuts. Proceedings of the Eighth IEEE International Conference on Computer Vision, ICCV 2001.

[6] Kernighan B.W., Lin S. (1970). An Efficient Heuristic Procedure for Partitioning Graphs. Bell Syst. Tech. J..

[7] Zachary W. (1977). An information Flow Model for Conflict and Fission in Small Groups. J. Anthropol. Res..

[8] White H.C., Boorman S.A., Breiger R.L. (1976). Social Structure from Multiple Networks. I. Blockmodels of Roles and Positions. Am. J. Sociol..

[9] Mason W.A., Conrey F.R., Smith E.R. (2007). Situating Social Influence Processes: Dynamic, Multidirectional Flows of Influence Within Social Networks. Personal. Soc. Psychol. Rev..

[10] Liu T., Ceder A.A. (2020). Battery-Electric Transit Vehicle Scheduling with Optimal Number of Stationary Chargers. Transp. Res. Part Emerg. Technol..

[11] Darvishan A., Lim G.J. (2021). Dynamic Network Flow Optimization for Real-Time Evacuation Reroute Planning Under Multiple Road Disruptions. Reliab. Eng. Syst. Saf..

[12] Dhamala T.N., Pyakurel U., Dempe S. (2018). A Critical Survey on the Network Optimization Algorithms for Evacuation Planning Problems. Int. J. Oper. Res..

[13] Pyakurel U., Dempe S., Kotsireas I.S., Nagurney A., Pardalos P.M., Tsokas A. (2021). Universal Maximum Flow with Intermediate Storage for Evacuation Planning. Dynamics of Disasters.

[14] Pyakurel U., Nath H.N., Dempe S., Dhamala T.N. (2019). Efficient Dynamic Flow Algorithms for Evacuation Planning Problems with Partial Lane Reversal. Mathematics.

[15] Temkin O.N., Zeigarnik A.V., Bonchev D. (2022). Chemical Reaction Networks: A Graph- Theoretical Approach.

[16] Rushdi A.M.A., Alsalami O.M. (2021). Reliability Analysis of Flow Networks with an Ecological Perspective. Netw. Biol..

[17] Chow W.-M. (1990). Assembly Line Design.

[18] Lighthill N.J., Whitham G.B. (1955). On Kinematic Waves. II. A Theory of Traffic Flow on Long Crowded Roads. Proc. R. Soc. Lond. Ser. A.

[19] Richards P.I. (1956). Shock Waves on the Highway. Oper. Res..

[20] Holden H., Risebro N.H. (1995). A Mathematical Model of Traffic Flow on a Network of Unidirectional Roads. SIAM J. Math. Anal..

[21] Haut B., Bastin G. (2007). A Second Order Model of Road Junctions in Fluid Models of Traffic Networks. Netw. Heterog. Media.

[22] Treiber M., Kesting A. (2013). Traffic Flow Dynamics: Data, Models, and Simulation.

[23] Scardoni G., Laudanna C. (2012). Identifying Critical Traffic Jam Areas with Node Centralities Interference and Robustness. Netw. Heterog. Media.

[24] Ahuja R.K., Magnanti T.L., Orlin J.B. (1993). Network Flows. Theory, Algorithms, and Applications.

[25] Garavello M., Piccoli B. (2009). On Fluido—Dynamic Models for Urban Traffic. Netw. Heterog. Media.

[26] Goatin P. (2009). Traffic Flow Models with Phase Transitions on Road Networks. Netw. Heterog. Media.

[27] Garavello M. (2010). A Review of Conservation Laws on Networks. Netw. Heterog. Media.

[28] Mouronte M.L., Benito R.M. (2015). Structural Analysis and Traffic Flow in the Transport Networks of Madrid. Netw. Heterog. Media.

[29] Bressan A., Nguyen K.T. (2015). Conservation Law Models for Traffic Flow on a Network of Roads. Netw. Heterog. Media.

[30] Herty M., Fazekas A., Visconti G. (2018). A Two-Dimensional Data-Driven Model for Traffic Flow on Highways. Netw. Heterog. Media.

[31] Herty M., Klar A. (2003). Modeling, Simulation, and Optimization of Traffic Flow Networks. SIAM J. Sci. Comput..

[32] Chiarello F.A., Goatin P. (2019). Non-local Multi-class Traffic Flow Models. Netw. Heterog. Media.

[33] Garavello M., Piccoli B. (2006). Traffic Flow on Networks.

[34] Nagatani T. (2002). The Physics of Traffic Jams. Rep. Prog. Phys..

[35] Ezaki T., Nishi R., Nishinari K. (2015). Taming Macroscopic Jamming in Transportation Networks. J. Stat. Mech. Theory Exp..

[36] Cordeau J.F., Toth P., Vigo D. (1998). A Survey of Optimization Models for Train Routing and Scheduling. Transp. Sci..

[37] Caimi G., Chudak F., Fuchsberger M., Laumanns M., Zenklusen R. (2011). A New Resource- Constrained Multicommodity Flow Model for Conflict-Free Train Routing and Scheduling. Transp. Sci..

[38] Arani A.M., Jolai F., Nasiri M.M. (2019). A Multi-Commodity Network Flow Model for Railway Capacity Optimization in Case of Line Blockage. Int. J. Rail Transp..

[39] Sun D., Strub I.S., Bayen A.M. (2007). Comparison of the Performance of Four Eulerian Network Flow Models for Strategic Air Traffic Management. Netw. Heterog. Media.

[40] Ng M.K., Chen C.H., Lee C.K. (2021). Mathematical Programming Formulations for Robust Airside Terminal Traffic Flow Optimisation Problem. Comput. Ind. Eng..

[41] Banda M.K., Herty M., Klar A. (2006). Gas Flow in Pipeline Networks. Netw. Heterog. Media.

[42] Gugat L., Herty M., Schleper V. (2011). Flow Control in Gas Networks: Exact Controllability to a Given Demand. Math. Methods Appl. Sci..

[43] Corbet T.F., Beyeler W., Wilson M.L., Flanagan T.P. (2018). A Model for Simulating Adaptive, Dynamic Flows on Networks: Application to Petroleum Infrastructure. Reliab. Eng. Syst. Saf..

[44] Rüffler F., Mehrmann V., Hante F.M. (2016). Optimal Model Switching for Gas Flow in Pipe Networks. Netw. Heterog. Media.

[45] Osiadacz A. (1984). Simulation of Transient Gas Flows in Networks. Int. J. Numer. Methods Fluids.

[46] Kiuchi T. (1994). An Implicit Method for Transient Gas Flows in Pipe Networks. Int. J. Heat Fluid Flow.

[47] Greyvenstein G.P. (2002). An Implicit Method for the Analysis of Transient Flows in Pipe Networks. Int. J. Numer. Methods Eng..

[48] Federgruen A., Groenevelt H. (1986). Optimal Flows in Networks with Multiple Sources and Sinks, with Applications to Oil and Gas Lease Investment Programs. Orepations Res..

[49] Gugat M., Hante F.M., Hirsch-Dick M., Leugering G. (2015). Stationary states in gas networks. Netw. Heterog. Media.

[50] Lämmer S., Kori H., Peters K., Helbing D. (2006). Decentralised Control of Material or Traffic Flows in Networks Using Phase-Synchronisation. Physica A.

[51] Donner R. (2008). Multivariate Analysis of Spatially Heterogeneous Phase Synchronisation in Complex Systems: Application to Self-Organised Control of Material Flows in Networks. Eur. Phys. J. B.

[52] Gugat M., Herty M., Klar A., Leugering G. (2005). Optimal Control for Traffic Flow Networks. J. Optim. Theory Appl..

[53] Herty M., Klar A. (2004). Simplified Dynamics and Optimization of Large Scale Traffic Networks. Math. Model. Methods Appl. Sci..

[54] Gugat M., Leugering G., Schmidt E.J.P.G. (2004). Global Controllability Between Steady Supercritical Flows in Channel Networks. Math. Methods Appl. Sci..

[55] Lämmer S., Helbing D. (2008). Self-Control of Traffic Lights and Vehicle Flows in Urban Road Networks. J. Stat. Mech. Theory Exp..

[56] Ajdari A. (2004). Steady Flows in Networks of Microfluidic Channels: Building on the Analogy with Electrical Circuit. Comptes Rendus Phys..

[57] Berli C.L.A. (2007). Theoretical Modelling of Electrokinetic Flow in Microchannel Networks. Colloids Surfaces A Physicochem. Eng. Asp..

[58] Bastin G., Bayen A.M., D’Apice C., Litrico X., Piccoli B. (2009). Open Problems and Research Perspectives for Irrigation Channels. Netw. Heterog. Media.

[59] Cantoni M., Weyer E., Li Y., Ooi S.K., Mareels I., Ryan M. (2007). Control of Large-Scale Irrigation Networks. Proc. IEEE.

[60] Mavkov B., Strecker T., Zecchin A.C., Cantoni M. (2022). Modeling and Control of Pipeline Networks Supplied by Automated Irrigation Channels. J. Irrig. Drain. Eng..

[61] Ferdowsi A., Valikhan-Anaraki M., Mousavi S.F., Farzin S., Mirjalili S. (2021). Developing a Model for Multi-Objective Optimization of Open Channels and Labyrinth Weirs: Theory and Application in Isfahan Irrigation Networks. Flow Meas. Instrum..

[62] Perez-Sanchez M., Sanchez-Romero F.J., Ramos H.M., Lopez-Jimenez P.A. (2016). Modeling Irrigation Networks for the Quantification of Potential Energy Recovering: A Case Study. Water.

[63] Kim H.-D., Kim J.-T., Nam W.-H., Kim S.-J., Choi J.-Y., Koh B.-S. (2016). Irrigation Canal Network Flow Analysis by a Hydraulic Mode. Irrig. Drain..

[64] Husain T., Abderrahman W.A., Khan H.U., Khan S.M., Khan A.U., Eqnaibi B.S. (1988). Flow Simulation Using Channel Network Model. J. Irrig. Drain. Eng..

[65] Labadie J.W. (2004). Optimal Operation of Multireservoir Systems: State-of-the-Art Review. J. Water Resour. Manag..

[66] Bigelow P.E., Benda L.E., Miller D.J., Burnett K.M. (2007). On Debris Flows, River Networks, and the Spatial Structure of Channel Morphology. For. Sci..

[67] Koplik J., Lasseter T.J. (1985). Two-Phase Flow in Random Network Models of Porous Media. Soc. Pet. Eng. J..

[68] Blunt M.J., Jackson M.D., Piri M., Valvatne P.H. (2002). Detailed Physics, Predictive Capabilities and Macroscopic Consequences for Pore-Network Models of Multiphase Flow. Adv. Water Resour..

[69] Blunt M.J. (2001). Flow in Porous Media—Pore-Network Models and Multiphase Flow. Curr. Opin. Colloid Interface Sci..

[70] Walski T.M. (1983). Technique for Calibrating Network Models. J. Water Resour. Plan. Manag..

[71] Ormsbee L.E., Lingireddy S. (1977). Calibrating Hydraulic Network Models. J. Am. Water Work. Assoc..

[72] Herty M., Izem N., Seaid M. (2019). Fast and Accurate Simulations of Shallow Water Equations in Large Networks. Comput. Math. Appl..

[73] Armbruster D., Degond P., Ringhofer C.A. (2006). Model for the Dynamics of Large Queuing Networks and Supply Chains. SIAM J. Appl. Math..

[74] D’Apice C., Göttlich S., Herty M., Piccoli B. (2010). Modeling, Simulation, and Optimization of Supply Chains: A Continuous Approach.

[75] Armbruster D., Marthaler D., Ringhofer C. (2003). Kinetic and Fluid Model Hierarchies for Supply Chains. Multiscale Model. Simul..

[76] Bretti G., D’Apice C., Manzo R., Piccoli B. (2007). A Continuum-Discrete Model for Supply Chains Dynamics. Netw. Heterog. Media.

[77] Helbing D., Lämmer D.S., Seidel T., Seba P., Platkowski T. (2004). Physics, Stability and Dynamics of Supply Networks. Phys. Rev. E.

[78] Herty M., Ringhofer C. (2007). Optimization for Supply Chain Models with Policies. Physica A.

[79] D’Apice C., Manzo R., Piccoli B. (2010). Existence of Solutions to Cauchy Problems for a Mixed Continuum-Discrete Model for Supply Chains and Networks. J. Math. Anal. Appl..

[80] Adhikari R.S., Aste N., Manfren M. (2012). Multi-Commodity Network Flow Models for Dynamic Energy Management—Smart Grid Applications. Energy Procedia.

[81] Eboli M., Proto A.N., Squillante M., Kacprzyk N.J. (2013). Financial Applications of Flow Network Theory. Advanced Dynamics Modeling of Economic and Social Systems.

[82] Russell A.H. (1970). Cash Flows in Networks. Manag. Sci..

[83] Rhys J.M.W. (1970). A Selection Problem of Shared Fixed Costs and Network Flows. Manag. Sci..

[84] Nagurney A., Siokos S. (1997). Financial Networks: Statics and Dynamics.

[85] Nagurney A., Cruz J. (2002). International Financial Networks with Intermediation: Modeling, Analysis, and Computations. Comput. Manag. Sci..

[86] Giudici P., Spelta A. (2016). Graphical Network Models for International Financial Flows. J. Bus. Econ. Stat..

[87] Gautier A., Granot F. (1995). Forest Management: A Multicommodity Flow Formulation and Sensitivity Analysis. Manag. Sci..

[88] Haghani A., Oh S.C. (1996). Formulation and Solution of a Multi-Commodity, Multi-Modal Network Flow Model for Disaster Relief Operations. Transp. Res. Part A Policy Pract..

[89] Lin Y.-K. (2007). On a Multicommodity Stochastic-Flow Network with Unreliable Nodes Subject to Budget Constraint. Eur. J. Oper. Res..

[90] Hu T.C. (1963). Multi-Commodity Network Flows. Oper. Res..

[91] Bellmore M., Vemuganti R.R. (1973). On Multi-Commodity Maximal Dynamic Flows. Oper. Res..

[92] Rothschild B., Whinston A. (1966). On Two Commodity Network Flows. Oper. Res..

[93] Salimifard K., Bigharaz S. (2022). The Multicommodity Network Flow Problem: State of the Art Classification, Applications, and Solution Methods. Oper. Res..

[94] Wang I.-L. (2018). Multicommodity Network Flows: A Survey, Part I: Applications and Formulations. Int. J. Oper. Res..

[95] Aldous D.J., Mc Diarmid C., Scott A. (2009). Uniform Multicommodity Flow through the Complete Graph with Random Edge-Capacities. Oper. Res. Lett..

[96] D’Apice C., Manzo R. (2006). A Fluid Dynamic Model for Supply Chains. Netw. Heterog. Media.

[97] Kennington J.L. (1978). A Survey of Linear Cost Multicommodity Network Flows. Oper. Res..

[98] Sakarovitch M. (1973). Two Commodity Network Flows and Linear Programming. Math. Program..

[99] Göttlich S., Herty M., Klar A. (2005). Network Models for Supply Chains. Commun. Math. Sci..

[100] D’Apice C., Manzo R., Piccoli B. (2006). Packet Flow on Telecommunication Networks. SIAM J. Math. Anal..

[101] Chen W.-K. (2003). Theory of Nets: Flows in Networks.

[102] Douligeris C., Mazumdar R. (1992). A Game Theoretic Perspective to Flow Control in Telecommunication Networks. J. Frankl. Inst..

[103] Onaga K. (1967). Optimum Flows in General Communication Networks. J. Frankl. Inst..

[104] Filipiak J. (1988). Modelling and Control of Dynamic Flows in Communication Networks.

[105] Marigo A. (2006). Optimal Traffic Distribution and Priority Coefficients for Telecommunication Networks. Netw. Heterog. Media.

[106] Lucas M.W. (2010). Network Flow Analysis.

[107] Marigo A. (2007). Equilibria for Data Networks. Netw. Heterog. Media.

[108] Formaggia L., Quarteroni A., Veneziani A. (2009). Cardiovascular Mathematics.

[109] Wang X., Delestre O., Fullana J.M., Saito M., Ikenaga Y., Matsukawa M., Lagree P.Y. (2012). Comparing Different Numerical Methods for Solving Arterial 1D Flows in Networks. Comput. Methods Biomech. Biomed. Eng..

[110] Nicosia S., Pezzinga G. (2007). Mathematical Models of Blood Flow in the Arterial Network. J. Hydraul. Res..

[111] Bianconi G., Zecchina R. (2008). Viable Flux Distribution in Metabolic Networks. Netw. Heterog. Media.

[112] Karolyi G., Scheuring I., Czaran T. (2002). Metabolic Network Dynamics in Open Chaotic Flow. Chaos Interdiscip. J. Nonlinear Sci..

[113] Li Z., Wang R.S., Zhang X.S. (2008). Mass Flow Model and Essentiality of Enzymes in Metabolic Networks. Lect. Notes Oper. Res..

[114] Beguerisse-Diaz M., Bosque G., Oyarzun D., Pico J., Barahona M. (2018). Flux-Dependent Graphs for Metabolic Networks. NPJ Syst. Biol. Appl..

[115] Banasiak J., Falkiewicz A., Namayanja P. (2016). Asymptotic State Lumping in Transport and Diffusion Problems on Networks with Applications to Population Problems. Math. Model. Methods Appl. Sci..

[116] Bertaglia G., Pareschi L. (2021). Hyperbolic Models for the Spread of Epidemics on Networks: Kinetic Description and Numerical Methods. ESAIM Math. Model. Numer. Anal..

[117] Todinov M.T. (2013). Flow Networks. Analysis and Optimization of Repairable Flow Networks, Networks with Disturbed Flows, Static Flow Networks and Reliability Networks.

[118] Rossvall M., Esquivel A.C., Lancichinetti A., West J.D., Lambiotte R. (2014). Memory in Network Flows and its Effects on Spreading Dynamics and Community Detection. Nat. Commun..

[119] Helbing D., Buzna L., Johansson A., Werner T. (2005). Self-organized Pedestrian Crowd Dynamics: Experiments, Simulations, and Design Solutions. Transp. Sci..

[120] Aronson J.E. (1989). A Survey of Dynamic Network Flows. Ann. Oper. Res..

[121] Bozhenyuk A.V., Gerasimenko E.M., Kacprzyk J., Naumovich I. (2017). Flows in Networks under Fuzzy Conditions.

[122] Banasiak J., Namayanja P. (2014). Asymptotic Behaviour of Flows on Reducible Networks. Netw. Heterog. Media.

[123] Pastor J.M., Garcia-Algarra J., Galeano J., Iriondo J.M., Ramasco J.J. (2015). A Simple and Bounded Model of Population Dynamics for Mutualistic Networks. Netw. Heterog. Media.

[124] Logak E., Passat I. (2016). An Epidemic Model with Nonlocal Diffusion on Networks. Netw. Heterog. Media.

[125] Fabio Camilli F., De Maio R., Tosin A. (2017). Transport of Measures on Networks. Netw. Heterog. Media.

[126] Corli A., di Ruvo L., Malaguti L., Rosini M.D. (2017). Traveling Waves for Degenerate Diffusive Equations on Networks. Netw. Heterog. Media.

[127] Sun Y., Work D.B. (2018). Error Bounds for Kalman Filters on Traffic Networks. Netw. Heterog. Media.

[128] Shen W. (2018). Traveling Wave Profiles for a Follow-the-Leader Model for Traffic Flow with Rough Road Condition. Netw. Heterog. Media.

[129] Chuang Y.-L., Chou T., D’Orsogna M.R. (2019). A Network Model of Immigration: Enclave Formation vs. Cultural Integration. Netw. Heterog. Media.

[130] Carlson R. (2014). Myopic Models of Population Dynamics on Infinite Networks. Netw. Heterog. Media.

[131] Ford L.R., Fulkerson D.R. (1958). Constructing Maximal Dynamic Flows from Static Flows. Oper. Res..

[132] Golberg A.V., Tarjan R.E. (1988). A New Approach to the Maximum-Flow Problem. J. Assoc. Comput. Mach..

[133] Megiddo N. (1974). Optimal Flows in Networks with Multiple Sources and Sinks. Math. Program..

[134] Wilkinson W.L. (1971). An Algorithm for Universal Maximal Dynamic Flows in a Network. Oper. Res..

[135] Philpott A.B. (1990). Continuous-Time Flows in Networks. Math. Oper. Res..

[136] Cherkassky B.V., Goldberg A.V., Radzik T. (1996). Shortest Paths Algorithms: Theory and Experimental Evaluation. Math. Program..

[137] Divoky J.J., Hung M.S. (1990). Performance of Shortest Path Algorithms in Network Flow Problems. Manag. Sci..

[138] Epstein D. (1998). Finding the k Shortest Paths. SIAM J. Comput..

[139] Ruhe G. (1991). Algorithmic Aspects of Flows in Networks.

[140] Williamson D.P. (2019). Network Flow Algorithms.

[141] Zheng Y.J., Chen S.Y. (2013). Cooperative Particle Swarm Optimization for Multiobjective Transportation Planning. Appl. Intell..

[142] Ringuest J.L., Rinks D.B. (1987). Interactive Solutions for the Linear Multiobjective Transportation Problems. Eur. J. Oper. Res..

[143] Edmonds J., Karp R.M. (1972). Theoretical Improvements in Algorithmic Efficiency for Network Flow Problems. J. Assoc. Comput. Mach..

[144] Lenstra J.K., Kan A.R. (1981). Complexity of Vehicle Routing and Scheduling Problems. Networks.

[145] Johnson D.B. (1977). Efficient Algorithms for Shortest Paths in Sparse Networks. J. Assoc. Comput. Mach..

[146] Climaco J.N., Antunes C.H., Alves M.J. (1993). Interactive Decision Support for Multiobjective Transportation Problems. Eur. J. Oper. Res..

[147] Meng Q., Lee D., Cheu R. (2005). Multiobjective Vehicle Routing and Scheduling Problem with Time Window Constraints in Hazardous Material Transportation. J. Transp. Eng..

[148] Gen M., Li Y.Z. (1998). Spanning Tree-Based Genetic Algorithm for Bicriteria Transportation Problem. Comput. Ind. Eng..

[149] Hamacher H.W., Pedersen C.R., Ruzika S. (2007). Multiple Objective Minimum Cost Flow Problems: A Review. Eur. J. Oper. Res..

[150] Ahuja R.K. (1986). Algorithms for the Minimax Transportation Problem. Nav. Res. Logist. Q..

[151] Bertsekas D.P. (1985). A Unified Framework for Primal-Dual Methods in Minimum Cost Network Flow Problems. Math. Program..

[152] Cunningham W.H., Frank A. (1985). A Primal-Dual Algorithm for Submodular Flows. Math. Oper. Res..

[153] Ahuja R.K., Orlin J.B. (1989). A Fast and Simple Algorithm for the Maximum Flow Problem. Oper. Res..

[154] Ahuja R.K., Batra J.L., Gupta S.K. (1984). A Parametric Algorithm for Convex Cost Network Flow and Related Problems. Eur. J. Oper. Res..

[155] Bertsekas D.P., Hosein P.A., Tseng P. (1987). Relaxation Methods for Network Flow Problems with Convex Arc Costs. SIAM J. Control Optim..

[156] Bertsekas D.P., Tseng P. (1988). Relaxation Methods for Minimum Cost Ordinary and Generalized Network Flow Problems. Oper. Res..

[157] Ali I., Charnes A., Tiantai S. (1985). Karmarkar’s Projective Algorithm: A Null Space Variant for Multi-Commodity Generalized Networks. Acta Math. Appl. Sin..

[158] Castro J. (2003). Solving Difficult Multicommodity Problems with a Specialized Interior-Point Algorithm. Ann. Oper. Res..

[159] Chardaire P., Lisser A. (2002). Simplex and Interior Point Specialized Algorithms for Solving Nonoriented Multicommodity Flow Problems. Oper. Res..

[160] Detlefsen N.K., Wallace S.W. (2002). The Simplex Algorithm for Multicommodity Networks. Netw. Int. J..

[161] Fleischer L., Sethuraman J. (2005). Efficient Algorithms for Separated Continuous Linear Programs: The Multicommodity Flow Problem with Holding Costs and Extensions. Math. Oper. Res..

[162] Assad A.A. (1978). Multicommodity Network Flows—A Survey. Networks.

[163] Dorneles A.P., de Araujo O.C., Buriol L.S. (2017). A Column Generation Approach to High School Timetabling Modeled as a Multicommodity Flow Problem. Eur. J. Oper. Res..

[164] Orlin J.B., Stein C. (1993). Parallel Algorithms for the Assignment and Minimum-Cost Flow Problems. Oper. Res. Lett..

[165] Anderson R.J., Setubal J.C. On the Parallel Implementation of Goldberg’s Maximum Flow Algorithm. Proceedings of the Fourth Annual ACM symposium on Parallel Algorithms and Architectures.

[166] Tseng P., Bertsekas D.P., Tsitsiklis J.N. (1990). Partially Asynchronous, Parallel Algorithms for Network Flow and Other Problems. SIAM J. Control Optim..

[167] Ciurea E., Ciupala L. (2004). Sequential and Parallel Algorithms for Minimum Flows. J. Appl. Math. Comput..

[168] Cheung T.Y. (1983). Graph Traversal Techniques and the Maximum Flow Problem in Distributed Computation. IEEE Trans. Softw. Eng..

[169] Kutija V. (1995). A Generalized Method for the Solution of Flows in Networks. J. Hydraul. Res..

[170] Reigstad G.A. (2015). Existence and Uniqueness of Solutions to the Generalized Riemann Problem for Isentropic Flow. SIAM J. Appl. Math..

[171] Bressan A., Yu F. (2015). Continuous Riemann Solvers for Traffic Flow at a Junction. Discret. Contin. Dyn. Syst..

[172] Colombo R.M., Garavello M. (2006). A Well Posed Riemann Problem for the P-system at a Junction. Netw. Heterog. Media.

[173] Contarino C., Toro E.F., Montecinos G.I., Borsche R., Kall J. (2016). Junction-Generalized Riemann Problem for Stiff Hyperbolic Balance Laws in Networks: An Implicit Solver and ADER schemes. J. Comput. Phys..

[174] Delle Monache M.L., Goatin P., Piccoli B. (2018). Priority-Based Riemann Solver for Traffic Flow on Networks. Commun. Math. Sci..

[175] Reigstad G.A., Flatten T., Haugen N.E., Ytrehus T. (2015). Coupling Constants and the Generalized Riemann Problem for Isothermal Junction Flow. J. Hyperbolic Differ. Equ..

[176] Bazaraa M.S., Jarvis J.J., Sherali H.D. (2005). Linear Programming and Network Flows.

[177] Baston V.J.D., Rahmouni M.K., Williams H.P. (1991). The Practical Conversion of Linear Programmes to Network Flow Models. Eur. J. Oper. Res..

[178] Dantzig G.B. (1998). Linear Programming and Extensions.

[179] Hobson E., Fletcher D.L., Stadlin W.O. (1984). Network Flow Linear Programming Techniques and Their Application to Fuel Scheduling and Contingency Analysis. IEEE Trans. Power Appar. Syst..

[180] Jewell W.S. (1962). New Methods in Mathematical Programming—Optimal Flow Through Networks with Gains. Oper. Res..

[181] Willekens F.J. (1999). Probability Models of Migration: Complete and Incomplete Data. SA J. Demogr..

[182] Blossfeld H.-P., Rohwer G. (2003). Techniques of Event History Modeling: New Approaches to Casual Analysis. J. R. Stat. Soc. Ser. D.

[183] Vitanov N.K., Vitanov K.N. (2018). On the Motion of Substance in a Channel of a Network and Human Migration. Physica A.

[184] Raymer J. (2007). The Estimation of International Migration Flows: A General Technique Focused on the Origin-Destination Association Structure. Environ. Plan. A.

[185] Greenwood M.J., Kemp-Leonard K. (2005). Modeling Migration. Encyclopedia of Social Measurement.

[186] Lee E.S. (1966). A Theory of Migration. Demography.

[187] Harris J.R., Todaro M.P. (1970). Migration, Unemployment and Development: A Two- Sector Analysis. Am. Econ. Rev..

[188] Simon J.H. (1999). The Economic Consequences of Immigration.

[189] Skeldon R. (1992). Migration and Development: A Global Perspective.

[190] Borjas G.J. (1989). Economic Theory and International Migration. Int. Migr. Rev..

[191] Vitanov N.K., Vitanov K.N. (2018). Discrete-time Model for a Motion of Substance in a Channel of a Network with Application to Channels of Human Migration. Physica A.

[192] Vitanov N.K., Vitanov K.N. (2019). Statistical Distributions Connected to Motion of Substance in a Channel of a Network. Physica A.

[193] Fawcet J.T. (1989). Networks, Linkages, and Migration Systems. Int. Migr. Rev..

[194] Gurak D.T., Caces F., Kitz M.M., Lim L.L., Zlotnik H. (1992). Migration Networks and the Shaping of Migration Systems. International Migration Systems: A Global Approach.

[195] Vitanov N.K., Vitanov K.N. (2016). Box Model of Migration Channels. Math. Soc. Sci..

[196] Vitanov N.K., Vitanov K.N., Ivanova T. (2018). Box Model of Migration in Channels of Migration Networks. Adv. Comput. Ind. Math..

[197] Vitanov N.K., Borisov R. (2018). A Model of a Motion of Substance in a Channel of a Network. J. Theor. Appl. Mech..

[198] Vitanov N.K., Borisov R. (2018). Statistical Characteristics of a Flow of Substance in a Channel of Network that Contains Three Arms. Stud. Comput. Intell..

[199] Borisov R., Vitanov N.K. (2019). Human Migration: Model of a Migration Channel with a Secondary and a Tertiary Arm. AIP Conf. Proc..

[200] Vitanov N.K., Dimitrova Z.I., Ausloos M. (2010). Verhulst-Lotka-Volterra Model of Ideological Struggle. Physica A.

[201] Vitanov N.K., Ausloos M., Rotundo G. (2012). Discrete Model of Ideological Struggle Accounting for Migration. Adv. Complex Syst..

[202] Vitanov N.K., Jordanov I.P., Dimitrova Z.I. (2009). On Nonlinear Dynamics of Interacting Populations: Coupled Kink Waves in a System of Two Populations. Commun. Nonlinear Sci. Numer. Simul..

[203] Vitanov N.K., Jordanov I.P., Dimitrova Z.I. (2009). On Nonlinear Population Waves. Appl. Math. Comput..

[204] Vitanov N.K., Dimitrova Z.I., Vitanov K.N. (2013). Traveling Waves and Statistical Distributions Connected to Systems of Interacting Populations. Comput. Math. Appl..

[205] Vitanov N.K., Vitanov K.N. (2013). Population Dynamics in Presence of State Dependent Fluctuations. Comput. Math. Appl..

[206] Schubert A., Glänzel W. (1984). A Dynamic Look at a Class of Skew Distributions. A Model With Scientometric Application. Scientometrics.

[207] Vitanov N.K. (2016). Science Dynamics and Research Production. Indicators, Indexes, Statistical Laws, and Mathematical Models.

[208] Johnson N., Kotz S. (1977). Urn Models and Their Applications. An Approach to Modern Discrete Probability Theory.

[209] Dietz K. (1966). On The Model of Weiss for the Spread of Epidemics by Carriers. J. Appl. Probab..

[210] Boucheron S., Gardy D. (1997). An Urn Model from Learning Theory. Random Struct. Algorithms.

[211] Kerner B.S. (2004). The Physics of Traffic.

[212] Bellomo N., Delitala M., Coscia V. (2002). On the Mathematical Theory of Vehicular Traffic Flow I. Fluid Dynamic and Kinetic Modelling. Math. Model. Methods Appl. Sci..

[213] Arlotti L., Bellomo N., De Angelis E. (2002). Generalized Kinetic (Boltzmann) models: Mathematical Structures and Applications. Math. Model. Methods Appl. Sci..

[214] Bonzani I. (2000). Hydrodynamic Models of Traffic Flow: Drivers’ Behaviour and Nonlinear Diffusion. Math. Comput. Model..

[215] Aw A., Klar A., Materne T., Rascle M. (2002). Derivation of Continuum Traffic Flow Models from Microscopic Follow-the-Leader Models. SIAM J. Appl. Math..

[216] Colombo R.M. (2002). Hyperbolic Phase Transitions in Traffic Flow. SIAM J. Appl. Math..

[217] De Angelis E. (1999). Nonlinear Hydrodynamic Models of Traffic Flow Modelling and Mathematical Problems. Math. Comput. Model..

[218] Treiber M., Kesting A., Helbing D. (2006). Delays, Inaccuracies and Anticipation in Microscopic Traffic Models. Physica A.

[219] Leutzbach W. (1988). Introduction to the Theory of Traffic Flow.

[220] Prigogine I., Herman R. (1971). Kinetic Theory of Vehicular Traffic.

[221] Helbing D., Parisi J., Stefan C., Müller S.C., Zimmermann W. (1998). From Microscopic to Macroscopic Traffic Models. A Perspective Look at Nonlinear Media.

[222] Ben-Naim E., Krapivsky P.L. (1998). Steady-State Properties of Traffic Flows. J. Phys. A.

[223] Ben-Naim E., Krapivsky P.L. (2003). Kinetic Theory of Traffic Flows. Traffic Granul. Flow.

[224] Günther M., Klar A., Materne T., Wegener R. (2002). An Explicitly Solvable Kinetic Model for Vehicular Traffic and Associated Macroscopic Equations. Math. Comput. Model..

[225] Klar A., Wegener R. (2000). Kinetic Derivation of Macroscopic Anticipation Models for Vehicular Traffic. SIAM J. Appl. Math..

[226] Helbing D. (2001). Traffic and Related Self-Driven Many-Particle Systems. Rev. Mod. Phys..

[227] Herty M., Kirchner C., Moutari S. (2006). Multi-Class Traffic Models on Road Networks. Commun. Math. Sci..

[228] Bellomo N. (2007). Modelling Complex Living Systems. A Kinetic Theory and Stochastic Game Approach.

[229] Coclite G.M., Garavello M., Piccoli B. (2005). Traffic Flow on a Road Network. SIAM J. Math. Anal..

[230] Dafermos C.M. (2005). Hyperbolic Conservation Laws in Continuum Physics.

[231] Bressan A. (2000). Hyperbolic Systems of Conservation Laws.

[232] Aw A., Rascle M. (2000). Resurection of “Second Order” Models of Traffic Flow. SIAM J. Appl. Math..

[233] Zhang H.M. (2002). A Non-equilibrium Traffic Model Devoid of Gas-like Behavior. Transp. Res. Part B Methodol..

[234] Shen C., Sun M. (2010). Formation of Delta Shocks and Vacuum States in the Vanishing Pressure Limit of Riemann Solutions to the Perturbed Aw–Rascle Model. J. Differ. Equ..

[235] Goatin P. (2006). The Aw–Rascle Vehicular Traffic Flow Model with Phase Transitions. Math. Comput. Model..

[236] Pan L., Han X. (2013). The Aw–Rascle Traffic Model with Chaplygin Pressure. J. Math. Anal. Appl..

[237] Dimarco G., Tosin A. (2010). The Aw–Rascle Traffic model: Enskog-Type Kinetic Derivation and Generalisations. J. Stat. Phys..

[238] Greenberg J.M. (2002). Extensions and Amplifications of a Traffic Model of Aw and Rascle. SIAM J. Appl. Math..

[239] Garavello M., Piccoli B. (2006). Traffic Flow on a Road Network Using the Aw– Rascle Model. Commun. Partial. Differ. Equ..

[240] Herty M., Rascle M. (2006). Coupling Conditions for a Class of Second-Order Models for Traffic Flow. SIAM J. Math. Anal..

[241] Colombo R.M., Marcellini F., Rascle M. (2010). A 2-phase Traffic Model Based on a Speed Bound. SIAM J. Appl. Math..

[242] Colombo R.M., Holden H., Marcellini F. (2020). On the Microscopic Modeling of Vehicular Traffic on General Networks. SIAM J. Appl. Math..

[243] Colombo R.M., Goatin P., Piccoli B. (2010). Road Networks with Phase Transitions. J. Hyperbolic Differ. Equ..

[244] Kerner B.S., Klenov S.L. (2002). A microscopic model for phase transitions in traffic flow. J. Phys. A Math. Gen..

[245] Kerner B.S., Klenov S.L. (2009). Phase Transitions in Traffic Flow on Multilane Roads. Phys. Rev. E.

[246] Fan S., Work D.B. (2015). A Heterogeneous Multiclass Traffic Flow Model with Creeping. SIAM J. Appl. Math..

[247] Blandin S., Work D., Goatin D.P., Piccoli B., Bayen A. (2011). A General Phase Transition Model for Vehicular Traffic. SIAM J. Appl. Math..

[248] D’Apice C., Manzo R., Piccoli B. (2008). A Fluid Dynamic Model for Telecommunication Networks with Sources and Destinations. SIAM J. Appl. Math..

[249] D’Apice C., Manzo R., Piccoli B. (2012). On the Validity of Fluid-Dynamic Models for Data Networks. J. Netw..

[250] Frost V.S., Melamed B. (1994). Traffic Modeling for Telecommunications Networks. IEEE Commun. Mag..

[251] Espitia N., Girard A., Marchand N., Prieur C. (2017). Fluid-Flow Modeling and Stability Analysis of Communication Networks. IFAC-PapersOnLine.

[252] Leugering G., Schmidt J. (2002). On The Modelling and Stabilization of Flows in Metworks of Open Canals. SIAM J. Control Optim..

[253] Gugat M. (2012). Contamination Source Determination in Water Distribution Networks. SIAM J. Appl. Math..

[254] Gugat M., Leugering G. (2003). Global Boundary Controllability of the De St. Venant Equations Between Steady States. Ann. L’IHP Anal. Non Linéaire.

[255] Colombo R.M., Herty L., Sachers V. (2002). On 2 × 2 Conservation Laws at a Junction. SIAM J. Math. Anal..

[256] Bressan A., Canic S., Garavello M., Herty M., Piccoli B. (2014). Flows on Networks: Recent Results and Perspectives. EMS Surv. Math. Sci..

[257] Herty M., Klar A., Piccoli B. (2007). Existence of Solutions for Supply Chain Models based on Partial Differential Equations. SIAM J. Math. Anal..

[258] Armbruster D., De Beer C., Freitag M., Jagalski T., Ringhofer C. (2006). Autonomous Control of production Networks using a Pheromone Approach. Physica A.

[259] Audenaert P., Colle D., Pickave M. (2019). Policy-Compliant Maximum Network Flows. Appl. Sci..

[260] Pyakurel U., Dempe S. (2020). Network Flow with Intermediate Storage: Models and Algorithms. SN Oper. Res. Forum.

[261] D’Apice C., Manzo R., Piccoli B. (2009). Modelling Supply Networks with Partial Differential Equations. Q. Appl. Math..

[262] Armbruster D., Degond P., Ringhofer C. (2007). Kinetic and Fluid Models for Supply Chains Supporting Policy Attributes. Bull. Inst. Math. Acad. Sin..

[263] Göttlich S., Herty M., Klar A. (2006). Modelling and Optimization of Supply Chains on Complex Networks. Commun. Math. Sci..

[264] Guo P., Sun Z., Peng C., Chen H., Ren J. (2019). Transient-Flow Modeling of Vertical Fractured Wells with Multiple Hydraulic Fractures in Stress-Sensitive Gas Reservoirs. Appl. Sci..

[265] Bretti G., Natalini R., Piccoli B. (2006). Numerical Approximations of a Traffic Flow Model on Networks. Netw. Heterog. Media.

[266] Buttazzo G., Santambrogio F. (2007). Asymptotical Compliance Optimization for Connected Networks. Netw. Heterog. Media.

[267] Bürger R., Garcia A., Karlsen K.H., Towers J.D. (2008). Difference Schemes, Entropy Solutions, and Speedup Impulse for an Inhomogeneous Kinematic Traffic Flow Model. Netw. Heterog. Media.

[268] Kurganov A., Polizzi A. (2009). Non-Oscillatory Central Schemes for Traffic Flow Models with Arrhenius Look-Ahead Dynamics. Netw. Heterog. Media.

[269] Tossavainen O.-P., Work D.B. (2013). Markov Chain Monte Carlo Based Inverse Modeling of Traffic Flows Using GPS Data. Netw. Heterog. Media.

[270] Reigstad G.A. (2014). Numerical Network Models and Entropy Principles for Isothermal Junction Flow. Netw. Heterog. Media.

[271] Herty M. (2007). Modeling, Simulation and Optimization of Gas Networks with Compressors. Netw. Heterog. Media.

[272] Holden H., Risebro N.S. (2018). Follow-the-Leader Models can be Viewed as a Numerical Approximation to the Lighthill-Whitham-Richards Model for Traffic Flow. Netw. Heterog. Media.

[273] Joly P., Kachanovska M., Semin A. (2019). Wave Propagation in Fractal Trees. Mathematical and Numerical Issues. Netw. Heterog. Media.

[274] Mantri Y., Herty M., Noelle S. (2019). Well-balanced Scheme for Gas-flow in Pipeline Networks. Netw. Heterog. Media.

[275] Qiu Y., Grundel S., Stoll M., Benner P. (2020). Efficient Numerical Methods for Gas Network Modeling and Simulation. Netw. Heterog. Media.

[276] Briani M., Cristiani E. (2014). An Easy-to-use Algorithm for Simulating Traffic Flow on Networks: Theoretical Study. Netw. Heterog. Media.

[277] Vitanov N.K., Dimitrova Z.I., Vitanov K.N. (2021). Simple Equations Method (SEsM): Algorithm, Connection with Hirota Method, Inverse Scattering Transform Method, and Several Other Methods. Entropy.

[278] Vitanov N.K. (2019). Recent Developments of the Methodology of the Modified Method of Simplest Equation with Application. Pliska Stud. Math. Bulg..

[279] Vitanov N.K. (2019). Modified Method of Simplest Equation for Obtaining Exact Solutions of non-linear Partial Differential Equations: History, recent development and studied classes of equations. J. Theor. Appl. Mech..

[280] Vitanov N.K. (2019). The Simple Equations Method (SEsM) For Obtaining Exact Solutions Of non-linear PDEs: Opportunities Connected to the Exponential Functions. AIP Conf. Proc..

[281] Vitanov N.K., Dimitrova Z.I. (2019). Simple Equations Method (SEsM) and Other Direct Methods for Obtaining Exact Solutions of non-linear PDEs. AIP Conf. Proc..

[282] Vitanov N.K. (2010). Application of Simplest Equations of Bernoulli and Riccati Kind for Obtaining Exact Traveling-Wave Solutions for a Class of PDEs with Polynomial non-linearity. Commun. Non-Linear Sci. Numer. Simul..

[283] Vitanov N.K. (2011). Modified Method of Simplest Equation: Powerful Tool for Obtaining Exact and Approximate Traveling-Wave Solutions of non-linear PDEs. Commun. Non-Linear Sci. Numer. Simul..

[284] Vitanov N.K. (2011). On Modified Method of Simplest Equation for Obtaining Exact and Approximate Solutions of non-linear PDEs: The Role of the Simplest Equation. Commun. Non-Linear Sci. Numer. Simul..

[285] Vitanov N.K. (2012). On Modified Method of Simplest Equation for Obtaining Exact Solutions of non-linear PDEs: Case of Elliptic Simplest Equation. Pliska Stud. Math. Bulg..

[286] Vitanov N.K., Dimitrova Z.I. (2018). Modified Method of Simplest Equation Applied to the non-linear Schrödinger Equation. J. Theor. Appl. Mech. Sofia.

[287] Jordanov I.P., Vitanov N.K. (2019). On the Exact Traveling Wave Solutions of a Hyperbolic Reaction- Diffusion Equation. Stud. Comput. Intell..

[288] Nikolova E.V., Chilikova-Lubomirova M., Vitanov N.K. (2021). Exact Solutions of a Fifth-Order Korteweg–de Vries–type Equation Modeling non-linear Long Waves in Several Natural Phenomena. AIP Conf. Proc..

[289] Vitanov N.K. (2021). Simple Equations Method (SEsM) and Its Connection with the Inverse Scattering Transform Method. AIP Conf. Proc..

[290] Vitanov N.K., Dimitrova Z.I. (2021). Simple Equations Method (SEsM) and Its Particular Cases: Hirota Method. AIP Conf. Proc..

[291] Vitanov N.K., Dimitrova Z.I., Vitanov K.N. (2021). On the Use of Composite Functions in the Simple Equations Method to Obtain Exact Solutions of non-linear Differential Equations. Computation.

[292] Borisov R., Dimitrova Z.I., Vitanov N.K. (2020). Statistical Characteristics of Stationary Flow of Substance in a Network Channel Containing Arbitrary Number of Arms. Entropy.

[293] Vitanov N.K., Vitanov K.N., Kantz H. (2020). On the Motion of Substance in a Channel of a Network: Extended Model and New Classes of Probability Distributions. Entropy.

[294] Katz L., Patil G.P. (1965). Unified Treatment of a Broad Class of Discrete Probability Distributions. Classical and Contagious Discrete Distributions.

[295] Johnson N.L., Kemp A.W., Kotz S. (2005). Univariate Discrete Distributions.

[296] Gurland J., Tripathi R.C., Patil G.P., Kotz S., Ord J.K. (1975). Estimation of Parameters on Some Extensions of the Katz Family of Discrete Distributions Involving Hypergeometric Functions. Statistical Distributions in Scientific Work, Vol. 1: Models and Structures.

[297] Yousry M.A., Srivastava R.C. (1987). The Hyper-Negative Binomial Distribution. Biom. J..

[298] Bardwell G.E., Crow E.L. (1964). A Two-Parameter Family of Hyper-Poisson Distributions. J. Am. Stat. Assoc..

[299] Sundt B., Jewell W.S. (1981). Further Results on Recursive Evaluation of Compound Distributions. ASTIN Bull..

[300] Willmot G.E. (1988). Sundt and Jewell’s Family of Discrete Distributions. ASTIN Bull..

[301] Ord J.K. (1967). Graphical Methods For a Class of Discrete Distributions. J. R. Stat. Soc. Ser. A.

[302] Ord J.K. (1967). On a System of Discrete Distributions. Biometrika.

[303] Ord J.K. (1972). Families of Frequency Distributions.

[304] Kemp A.W. (1968). A Wide Class of Discrete Distributions and the Associated Differential Equations. Sankhya Ser. A.

[305] Dacey M.F. (1972). A Family of Discrete Probability Distributions Defined by the Generalized Hypergeometric Series. Sankhya Ser. B.

[306] Chakraborty S. (2015). Generating Discrete Analogues of Continuous Probability Distributions—A Survey of Methods and Constructions. J. Stat. Distrib. Appl..

[307] Alzaatreh A., Lee C., Famoye F. (2012). On the Discrete Analogues of Continuous Distributions. Stat. Methodol..

[308] Vitanov N.K., Borisov R., Vitanov K.N. (2021). On the Motion of Substance in a Channel and Growth of Random Networks. Physica A.

[309] Newman M. (2018). Networks.

[310] Krapivsky P.L., Redner S., Leyvraz F. (2000). Connectivity of Growing Random Networks. Phys. Rev. Lett..

[311] Krapivsky P.L., Redner S. (2001). Organization of Growing Random Networks. Phys. Rev. E.

